# Top‐down stepwise refinement identifies coding and noncoding RNA‐associated epigenetic regulatory maps in malignant glioma

**DOI:** 10.1111/jcmm.17244

**Published:** 2022-02-22

**Authors:** Yutao Huang, Xiangyu Gao, Erwan Yang, Kangyi Yue, Yuan Cao, Boyan Zhao, Haofuzi Zhang, Shuhui Dai, Lei Zhang, Peng Luo, Xiaofan Jiang

**Affiliations:** ^1^ Department of Neurosurgery Xijing Hospital Fourth Military Medical University Xi’an China; ^2^ State Key Laboratory of Cancer Biology Fourth Military Medical University Xi’an China

**Keywords:** biomarker, Cox‐Lasso regression, cross‐talk signalling, epigenetic regulation, integrative analysis, malignant glioma, noncoding RNA

## Abstract

With the emergence of the molecular era and retreat of the histology epoch in malignant glioma, it is becoming increasingly necessary to research diagnostic/prognostic/therapeutic biomarkers and their related regulatory mechanisms. While accumulating studies have investigated coding gene‐associated biomarkers in malignant glioma, research on comprehensive coding and noncoding RNA‐associated biomarkers is lacking. Furthermore, few studies have illustrated the cross‐talk signalling pathways among these biomarkers and mechanisms in detail. Here, we identified DEGs and ceRNA networks in malignant glioma and then constructed Cox/Lasso regression models to further identify the most valuable genes through stepwise refinement. Top‐down comprehensive integrated analysis, including functional enrichment, SNV, immune infiltration, transcription factor binding site, and molecular docking analyses, further revealed the regulatory maps among these genes. The results revealed a novel and accurate model (AUC of 0.91 and C‐index of 0.84 in the whole malignant gliomas, AUC of 0.90 and C‐index of 0.86 in LGG, and AUC of 0.75 and C‐index of 0.69 in GBM) that includes twelve ncRNAs, 1 miRNA and 6 coding genes. Stepwise logical reasoning based on top‐down comprehensive integrated analysis and references revealed cross‐talk signalling pathways among these genes that were correlated with the circadian rhythm, tumour immune microenvironment and cellular senescence pathways. In conclusion, our work reveals a novel model where the newly identified biomarkers may contribute to a precise diagnosis/prognosis and subclassification of malignant glioma, and the identified cross‐talk signalling pathways would help to illustrate the noncoding RNA‐associated epigenetic regulatory mechanisms of glioma tumorigenesis and aid in targeted therapy.

## 1 INTRODUCTION

Malignant glioma, the most common primary malignant tumour of the central nervous system, usually occurs in the sixth through eighth decades of life,^1^ has an annual incidence of 5.26 cases per 100,000 people and usually results in quick fatality.^2^, ^3^, ^4^ Malignant glioma basically consists of low‐grade glioma (LGG, WHO grades II–III) and glioblastoma multiform (GBM, WHO grade IV).^3^, ^5^, ^6^ Nearly, all LGGs will ultimately progress to GBM.^7^ The current therapy for newly diagnosed malignant glioma is surgical removal of the maximum safe amount of tumour followed by adjuvant radiation therapy and temozolomide chemotherapy.^8^ This approach has a 2‐year survival rate of 27% for newly diagnosed GBM, but the overall prognosis remains poor.^1^, ^2^ Therefore, it is urgent to elucidate the mechanisms of malignant glioma and find new treatments. Once upon a time, malignant gliomas have ever been classified, diagnosed and therapized based on histological characteristics and resemblance with a supposed cell type of origin.^9^ In the past decade, however, the classification, diagnosis and therapy of glioma have dramatically changed. Deletions involving chromosomes 1p/19q, IDH mutations, and so on are closely related to prognosis.^10^, ^11^, ^12^, ^13^ The latest guide incorporates molecular parameters, in addition to histology, into the classification, diagnosis and therapy of gliomas, contributing to a profound and in‐depth molecular era.^3^

While accumulating studies have investigated coding gene‐associated biomarkers in malignant glioma, research on comprehensive coding and noncoding RNA (ncRNA)‐associated biomarkers is lacking. Noncoding RNAs, which are an emerging class of transcripts that are encoded by the genome but are mostly not translated into proteins, consist of housekeeping RNAs, small ncRNAs (sncRNAs, including microRNAs, miscRNAs, circular RNAs and piRNAs, etc.), and long ncRNAs (lncRNAs).^14^, ^15^ sncRNAs and lncRNAs were once indicated as byproducts of the splicing procedure, until some researchers found that they may function in post‐transcriptional modification, the organization of nuclear domains, and the regulation of proteins or RNA molecules, indicating that the biological functions of ncRNAs are more pervasive than previously suspected.^16^, ^17^, ^18^, ^19^, ^20^, ^21^ Therefore, more research on these ncRNAs will lead to a greater understanding of cancer cell functions and may lead to novel clinical applications in oncology.^15^ The competing endogenous RNA (ceRNA) mechanism is one of the most important mechanisms of ncRNAs and indicates that endogenous coding and noncoding RNAs may share common microRNA‐binding sites and thus indirectly regulate the expression of each other by competing for microRNA binding.^22^ Researchers have focused on lncRNA/miRNA/mRNA‐associated ceRNA regulatory networks. However, ceRNA networks are not limited to lncRNA‐associated networks; they apply to all ncRNA‐associated networks.^22^ Therefore, it is necessary and valuable to mine the comprehensive ncRNA‐associated regulatory networks in glioma.

The emerging and accumulating applications of high‐throughput sequencing make it easier to research and identify differentially expressed genes (DEGs) or biomarkers of various tumours. Based on bioinformatics methods, DEGs can be used to further mine critical signalling pathways or molecular mechanisms to provide guidance or illuminate tumour research questions. However, previous bioinformatics studies on glioma usually identified biomarkers or performed functional enrichment analyses. Few studies have illustrated the cross‐talk signalling pathways among these biomarkers and mechanisms in detail. Illustration of the cross‐talk signalling pathways among biomarkers and mechanisms is beneficial for understanding glioma tumorigenesis and malignant progression, as well as promoting glioma therapy.

**FIGURE 1:**
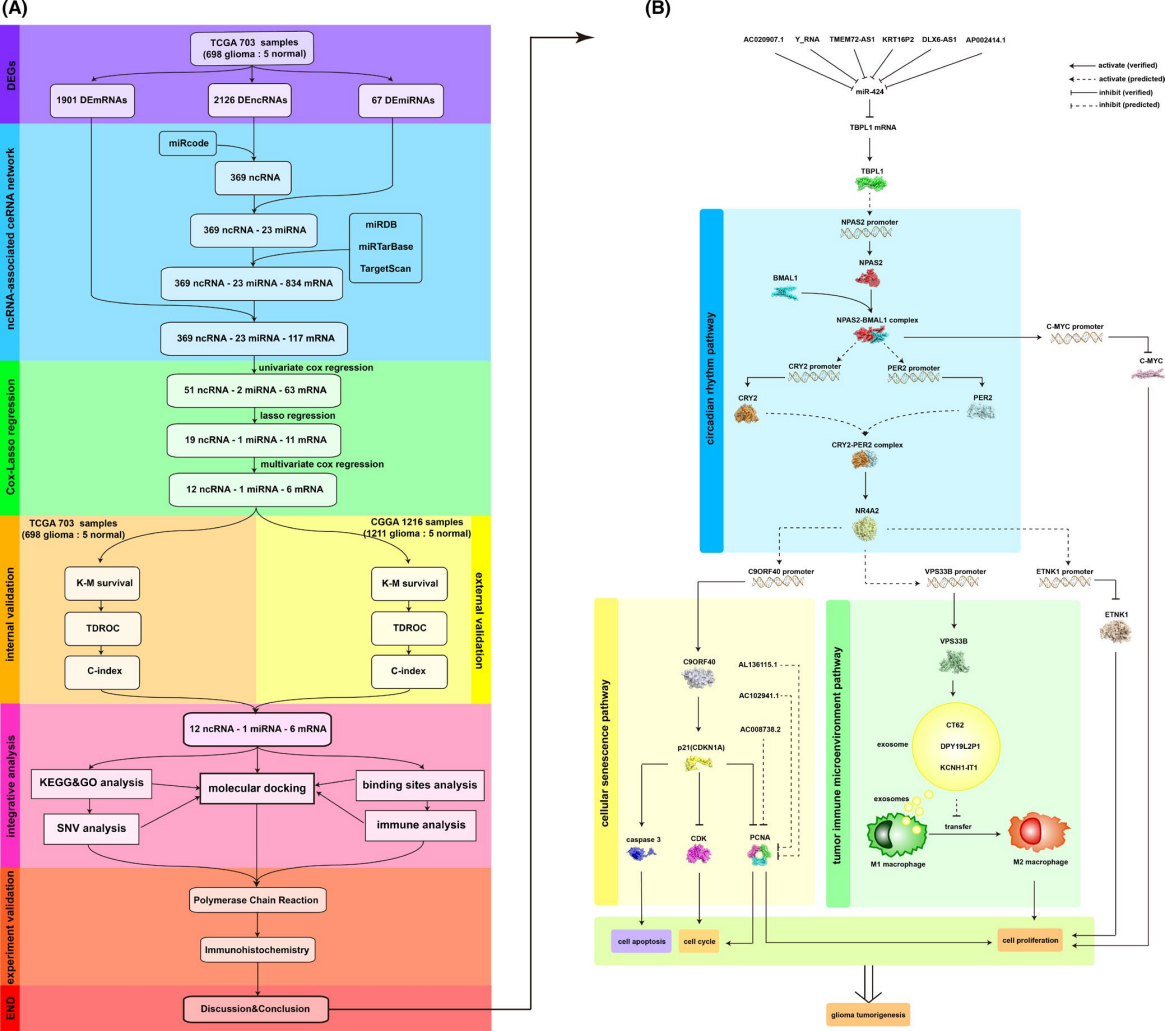
Algorithm tree and Cross‐talk signalling pathways. (A) Algorithm tree of the top‐down stepwise refinement method. TCGA, The Cancer Genome Atlas; CGGA, Chinese Glioma Genome Atlas; DEGs, differentially expressed genes; DEmRNAs, differentially expressed mRNAs; ncRNA, noncoding RNA; DEncRNAs, differentially expressed ncRNAs; DEmiRNAs, differentially expressed miRNAs; ceRNA, competing endogenous RNA; miRcode, miRcode database; miRDB, miRDB database; miRTarBase, miRTarBase database; TargetScan, TargetScan database; K‐M survival, Kaplan–Meier survival; TDROC, time‐dependent receiver operating characteristic; C‐index, concordance index; KEGG, Kyoto Encyclopedia of Genes and Genomes; GO, Gene Ontology; SNV, single nucleotide variation. (B) Cross‐talk signalling pathways and molecular mechanisms that involve the identified noncoding RNA‐associated biomarkers

‘Stepwise refinement’ was proposed by Swedish computer scientist Wirth in the 1970s and refers to compiling executable programs not in one step but in several steps and gradual refinement, where the program compiled in the first step is the most abstract, the program compiled in the second step is less abstract, and the program compiled in the last step is executable.^23^ Here, we used two databases, The Cancer Genome Atlas (TCGA) database, whose samples were derived mainly from America (698 malignant glioma samples and 5 normal brain samples), and the Chinese Glioma Genome Atlas (CGGA) database, whose samples were derived mainly from Asia (1211 malignant glioma samples and 5 normal brain samples), to produce, validate and mine the ncRNA‐associated networks. After the DEGs were identified, ncRNA‐associated ceRNA networks were constructed. Subsequently, Cox and Lasso regression models were used to further identify the most valuable genes in glioma tumorigenesis and then validate them in LGG and GBM. ‘Top‐down’ refers to dividing complex and large problems into small problems, determining the key points, and then qualitatively and quantitatively describing the problems with accurate thinking. Here, logical deduction based on references and top‐down comprehensive integrative analysis, including Gene Ontology (GO) and Kyoto Encyclopedia of Genes and Genomes (KEGG) functional enrichment, single nucleotide variation (SNV), immune infiltration, transcription factor binding site and molecular docking analyses, revealed that these genes were correlated with the circadian rhythm, tumour immune microenvironment, and cellular senescence pathways (Figure 1A,B). The epigenetic regulatory maps and signalling pathways among these genes were deduced and are discussed in the discussion section. This research aimed to determine the potential genes involved in glioma tumorigenesis and malignant progression and to illustrate the cross‐talk signalling pathways among these genes and molecular mechanisms based on references and comprehensive integrative analysis. As a result, our work reveals a novel model in which the newly identified biomarkers would contribute to a precise diagnosis/prognosis and subclassification of malignant glioma, and the identified cross‐talk signalling pathways would help to illustrate the noncoding RNA‐associated epigenetic regulatory mechanisms of glioma tumorigenesis and malignant progression and aid in targeted therapy.

## 2 MATERIALS AND METHODS

### 2.1 Data acquisition

The mRNA sequencing data, miRNA sequencing data, SNV data and corresponding clinical data used to construct the critical gene network and Cox/Lasso model in malignant glioma were obtained from the TCGA in the National Institutes of Health ‐ National Cancer Institute GDC data portal (https://portal.gdc.cancer.gov/, data release 26.0—8 September 2020). Data included 698 malignant glioma samples (529 GBM samples and 169 LGG samples) and 5 normal brain samples. The sequencing data were derived from the Illumina HiSeq platform (Illumina, Inc.) The research was conducted by the guidelines provided by the TCGA (http://cancergenome.nih.gov/publications/publicationguidelines).

The mRNA sequencing data, miRNA sequencing data and corresponding clinical data for validating the Cox and Lasso models were obtained from the CGGA (http://www.cgga.org.cn/, data release—14 June 2020). Data included 1211 malignant glioma samples (733 LGG samples and 478 GBM samples) and 5 normal brain samples. The mRNA sequencing data were derived from the Illumina HiSeq 2,000/2,500/4,000 Sequencing System, while the miRNA sequencing data were derived from the Agilent G2565BA Microarray Scanner System.

### 2.2 Extraction of DEGs and construction of the ncRNA‐miRNA‐mRNA network

The sequenced genes were mapped to the human genome reference (Homo_sapiens.GRCh38.101.chr) from the Ensembl database (https://asia.ensembl.org/index.html). The mRNAs were recognized and extracted using the keyword ‘protein_coding’, while the ncRNAs (non‐miRNA) were recognized and extracted using the following keywords (see supplementary material section Ⅰ).

The differentially expressed mRNAs (DEmRNAs) and differentially expressed ncRNAs (DEncRNAs) (non‐miRNA) were identified and depicted using the Linear Models for Microarray and RNA‐Seq Data (LIMMA) package in R software (version 3.5.1).^24^, ^25^, ^26^ The differentially expressed miRNAs (DEmiRNAs) were identified and depicted using the Empirical Analysis of Digital Gene Expression Data (edgeR) package in R software.^27^ The DEGs were identified using |log2(fold change)| (|log2FC|), which was set to 2, and the false discovery rate (FDR), which was set to 0.05.

The DEncRNAs (non‐miRNA) and DEmiRNAs were mapped to the ncRNA‐miRNA network in the miRcode database (http://www.mircode.org/) to identify the DEncRNAs (non‐miRNA) and DEmiRNAs in the ceRNA network. The target mRNAs were identified through the miRDB database,^28^ miRTarBase database^29^ and TargetScan database.^30^, ^31^ The ncRNA‐miRNA‐mRNA network was depicted using Cytoscape software^32^ (version 3.6.1).

### 2.3 Cox and Lasso regression models

First, the DEGs in the ncRNA‐miRNA‐mRNA network were added to the univariate Cox proportional hazards model (Cox) using the ‘survival’ package in R software (version 3.5.1). The Cox model was constructed as follows:

$$h\left(t,X_{i}\right)=h_{0}\left(t\right)\times \exp\left(X_{i}\cdot \beta \right),$$

where $h_{0}\left(t\right)$ is the benchmark risk equation, which can be any non‐negative equation for time; $X_{i}$ is the eigenvector of instance *i*; and *β* is the parameter vector, which is obtained by maximizing the Cox partial approximation.

Subsequently, the results were incorporated into the least absolute shrinkage and selection operator (Lasso) regression model using the ‘glmnet’ and ‘survival’ packages in R software (version 3.5.1). Given the objective function

$$\frac{1}{N}\sum_{i=1}^{N}f(x_{i},y_{i},\alpha ,\beta ),$$

the Lasso regression model would be constructed as follows:

$$\min_{\alpha ,\beta }\frac{1}{N}\sum_{i=1}^{N}{f\left(xi,yi,\alpha ,\beta \right)\;\text{subject}\;\text{to}\;||\beta ||}_{1}\leq t,$$

where only *β* is penalized, while *α* is free to take any allowed value.

Finally, the results were incorporated into the multivariate Cox model using the ‘survival’ package in R software (version 3.5.1) to further filter the most valuable genes. The patients’ risk scores were calculated and used to group patients into the high‐risk group and low‐risk group by the median using the ‘predict’ function of the ‘survival’ package in R software (version 3.5.1) based on gene expression in the model. Specifically speaking, if a gene is downregulated in malignant glioma and represents a worse prognosis, then this gene would be recognized as a useful gene in the multivariate Cox model (and obtains a regression coefficient; the same below), and the patients with this gene expression lower than the control would obtain a positive number score (where the concrete score is related to the gene expression level; the same below), while the patients with this gene expression higher than the control would obtain a negative number score. And if a gene is upregulated in malignant glioma and represents a worse prognosis, then this gene would be recognized as a useful gene in the model, and the patients with this gene expression higher than the control would obtain a positive number score, while the patients with this gene expression lower than the control would obtain a negative number score. Finally, after testing each gene expression, every patient would obtain a risk score which is calculated by the summation of the scores of each gene. Half of the patients (whose risk scores are higher than the median) would be categorized into the high‐risk group, and the others would be categorized into the low‐risk group.

The final model was evaluated through the internal (TCGA) and external (CGGA) datasets by Kaplan–Meier survival analysis, which was performed and depicted using the ‘survival’ and ‘survminer’ packages in R software (version 3.5.1). The TDROC curve was generated and depicted using the ‘survivalROC’ package in R software (version 3.5.1), and the C‐index was determined using the ‘survcomp’ package in R software (version 3.5.1). The area under the TDROC curve (AUC) was calculated as follows:

$$\text{AUC}\left(f\right)=\frac{\sum_{t_{0}\in D^{0}}\sum_{t_{1}\in D^{1}}1[f\left(t_{0}\right)<f\left(t_{1}\right)]}{\left|D^{0}\left|\cdot \right|D^{1}\right|},$$

where $1\left[f\left(t_{0}\right)<f\left(t_{1}\right)\right]$ denotes an indicator function that returns 1 if $f\left(t_{0}\right)<f\left(t_{1}\right)$ and 0 otherwise; $D^{0}$ is the set of negative examples; and $D^{1}$ is the set of positive examples.

The C‐index was calculated as follows:

$$\frac{1}{M}\sum_{i=\delta_{i}=1}\sum_{j=T_{i}<T_{j}}I[S\left(T_{i},X_{i}\right)<S\left(T_{j},X_{j}\right)],$$

where the function I[C] refers to I[C] = 1 if C is true; otherwise, I[C] = 0. The first summation function $\Sigma i:\delta_{i}=1$ indicates that at least one instance of the pair has an event. The second summation function $\Sigma j:T_{i}<T_{j}$ represents that the recording time Tj of another pair must be longer than the first instance event time. The two summation functions select all pair combinations that can be used for the comparison.

### 2.4 SNV analysis

The median was used to divide patients into the high‐risk group and low‐risk group based on gene expression in the model through the ‘predict’ function of the ‘survival’ package in R software (version 3.5.1). SNV analysis was performed and depicted using the ‘maftools’ package (https://bioconductor.org/packages/release/bioc/html/maftools.html) in R software (version 3.5.1).

### 2.5 Functional enrichment analysis

The functional enrichment analyses via the GO and KEGG databases were performed and depicted using the ‘clusterProfiler’, ‘org. Hs.eg.db’, ‘enrichplot’ and ‘ggplot2’ packages in R software (version 3.5.1) to identify the main biological functions of the genes in the model. The enrichment functions were identified using the adjusted p‐value, which was set to 0.05.

### 2.6 Analysis of infiltrating immune cells in malignant glioma

‘The Estimation of Stromal and Immune Cells in Malignant Tumor Tissues Using Expression Data’ (ESTIMATE, https://r‐forge.r‐project.org/projects/estimate/) package in R software (version 3.5.1) was used to predict tumour purity and the presence of infiltrating stromal/immune cells in glioma tissue. The correlation between the risk score and the immune filtration score was calculated and depicted using the ‘ggplot2’, ‘openxlsx’, ‘ggpubr’ and ‘ggExtra’ packages in R software (version 3.5.1). The proportion of each kind of immune cell in malignant glioma tissues based on various gene expression levels or risk scores was calculated and depicted using the ‘ggplot2’, ‘ggthemes’, ‘ggpubr’ and ‘ggcorrplot’ packages in R software (version 3.5.1).

### 2.7 Molecular docking analysis

RNA sequences were obtained from the NIH genetic sequence database (GenBank),^33^ while protein sequences were obtained from the UniProt database.^34^ The minimum free energy (MFE) secondary structures of the RNAs were constructed using a loop‐based energy model and the dynamic programming algorithm.^35^ The three‐dimensional structures of the RNAs were constructed based on the MFE secondary structure using the two‐step procedure^36^ and distance geometry (DG) method.^37^ The three‐dimensional structures of the RNAs were evaluated using all‐heavy‐atom knowledge‐based statistical potential (3dRNAscore).^38^ The three‐dimensional structures of the proteins were obtained from the worldwide Protein Data Bank (PDB) database (https://www.wwpdb.org/) if they were known; otherwise, they were constructed using remote homology detection methods.^39^ The interactions between RNAs and proteins were evaluated using the hybrid docking algorithm of template‐based modelling and free docking (HDOCK).^40^ The interactions between RNAs or proteins were evaluated using the docking energy score in HDOCK. The score used to evaluate the interaction between RNAs was set to −200, which means that a docking energy less than −200 could be recognized as a stabilized interactive conformation. Similarly, the docking energy score used to evaluate the interaction between proteins or RNAs and proteins was set to −300. The results were depicted using PyMOL software (The PyMOL Molecular Graphics System, Version 2.3.0 Open‐Source, Schrodinger LLC).

### 2.8 Transcription factor binding site analysis

The 2000 bp sequences upstream of the 5’‐untranslated region (5’‐UTR) are recognized as the potential sequences where promoters are located. The potential sequences where promoters are located were obtained from the National Center of Biotechnology Information (NCBI). Transcription factor binding sites were predicted using JASPAR software.^41^ The possibility of binding (relative score) was set to 0.9.

### 2.9 Tissue samples collection

The glioma tissues and corresponding adjacent tissues (NC) were collected from the patients who underwent surgery at Xijing Hospital, Fourth Military Medical University. All the tissue samples that were obtained from the operation room were immediately frozen and then stored in liquid nitrogen until RNA and protein detection or staining. All the tissues were confirmed and graded by the department of pathology of Xijing Hospital, Fourth Military Medical University, according to the WHO stage guideline.

### 2.10 Reverse‐transcription and quantitative real‐time polymerase chain reaction

The RNAiso plus (TAKARA) was used for isolating high purity of total RNA from tissues. RNA yields were determined with Nanodrop (Thermo Fisher Scientific). For microRNA reverse‐transcription analysis, the PrimeScript^TM^ RT reagent Kit (TAKARA) was used, and for other non‐microRNA reverse‐transcription analysis, the PrimeScript^TM^ RT Master Mix (TAKARA, Kusatsu, Japan) was used. The stem‐loop method was used to detect microRNA, and the procedure for microRNA reverse‐transcription was (1) 42℃ for 15 min, (2) 85℃ for 5 s, (3) 4℃ for +∞; and the process for non‐microRNA reverse‐transcription was (1) 37℃ for 15 min, (2) 85℃ for 5 s, (3) 4℃ for +∞. For qRT‐PCR, the SYBR Fast qPCR Mix (TAKARA) was used. The program for qRT‐PCR was 1), Reps: 1, 95℃ for 30 s; (2), Reps: 40, 95℃ for 5 s, 60℃ for 30 s; (3), Reps:1, 95℃ for 15 s, 60℃ for 60 s. Relative expression of RNA was determined using the 2^−△△CT^ method. All Ct values were normalized to glyceraldehyde 3‐phosphate dehydrogenase mRNA or vampyrus U6 spliceosomal RNA. The primer sequences were given in Supplementary Table 1.

### 2.11 Immunohistochemistry (IHC)

The slides were retrieved by the method of heat‐induced epitope retrieval and then blocked in 5% goat serum (LOT NO: SP‐9001, ZSGB‐bio) for 10 min. Then the primary antibody (anti‐TBPL1 antibody, LOT NO: PA5‐28088, 2 μg/ml, Invitrogen) was added and incubated with gentle agitation overnight at 4°C. After washing with PBS three times, the secondary antibody working solution (Goat Anti‐Rabbit IgG H&L, HRP, LOT NO: SP‐9001, ZSGB‐bio) was subsequently added and incubated for 10 min at room temperature. The slides were washed with PBS three times, and then the Streptavidin/HRP working solution (LOT NO: SP‐9001, ZSGB‐bio) was added and incubated for 10 min at room temperature. After washing with PBS three times, the fresh DAB working solution (LOT NO: SP‐9001, ZSGB‐bio) was added and incubated for 5 min at room temperature. The slides were washed, haematoxylin stained, and finally observed under the inverted fluorescence microscopy (Olympus IX73).

### 2.12 Statistical analysis

The interactions between RNAs or proteins were evaluated using the docking energy score in HDOCK. The score used to evaluate the interaction between RNAs was set to −200, which means that a docking energy less than −200 could be recognized as a stabilized interactive conformation. Similarly, the docking energy score used to evaluate the interaction between proteins or RNAs and proteins was set to −300. The DEGs were identified using |log2(fold change)| (|log2FC|), which was set to 2, and the FDR, which was set to 0.05. The enrichment functions were identified using the adjusted *p*‐value, which was set to 0.05. Patients were grouped into the high‐risk group and low‐risk group using the median. All statistical analyses were performed using R software (version 3.5.1).

### 2.13 Ethics approval

The glioma tissues and corresponding adjacent brain tissues were collected from the patients who underwent surgery at Xijing Hospital, Fourth Military Medical University. All the obtained tissues were approved by the Ethics Committee of Xijing Hospital, Fourth Military Medical University. The written informed consent was obtained from all patients or their relatives.

## 3 RESULTS

### 3.1 The DEGs and ceRNA network potentially involved in malignant glioma tumorigenesis

#### 3.1.1 DEGs in malignant glioma

**FIGURE 2:**
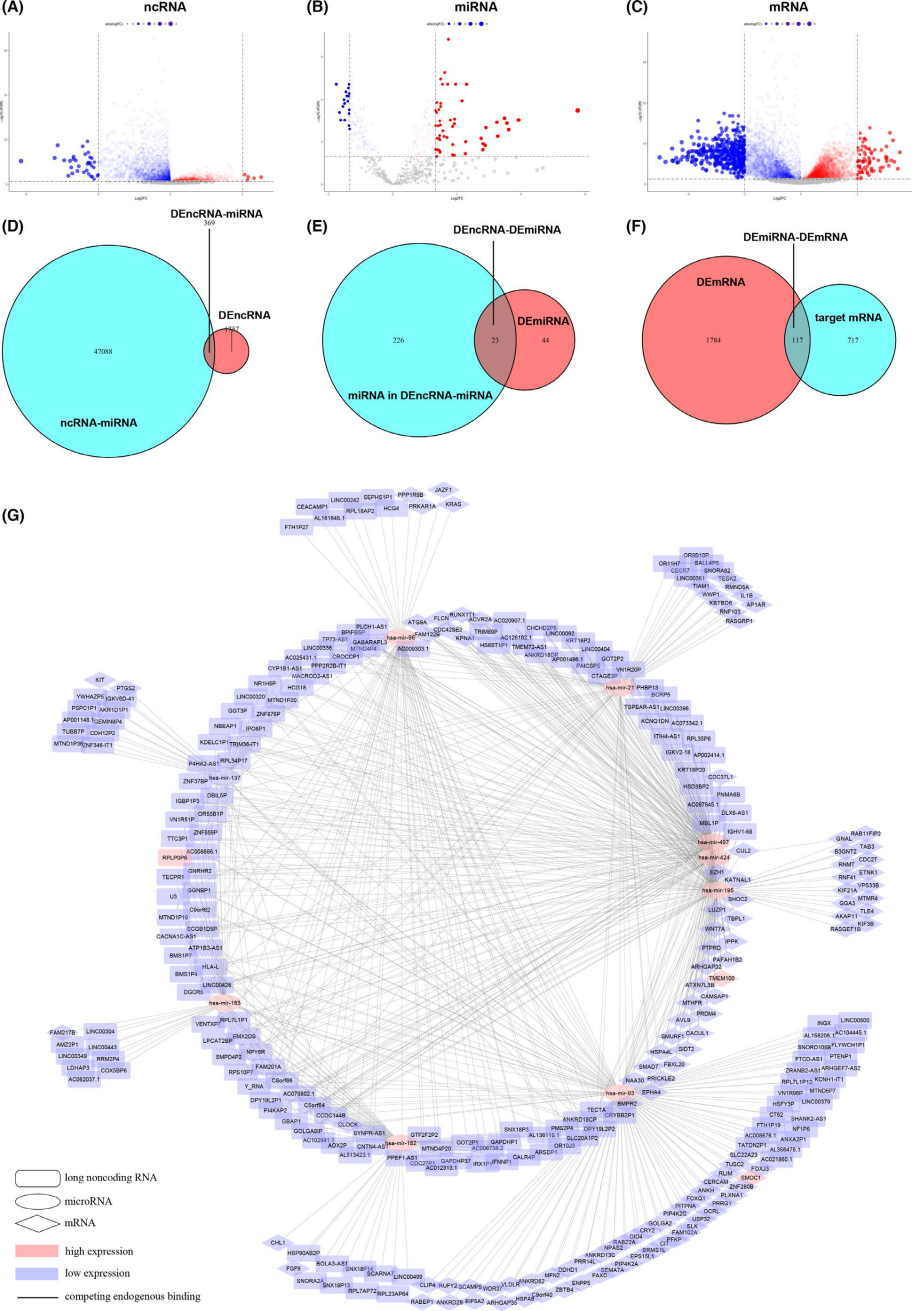
Potential DEGs and the ceRNA network in malignant glioma tumorigenesis and progression. Volcano plot of the differentially expressed (A) ncRNAs, (B) miRNAs and (C) mRNAs between malignant glioma and normal brain tissues; blue dots represent downregulated RNAs, and red dots represent upregulated RNAs; FC, fold change; FDR, false discovery rate. (D) Venn diagram of the differentially expressed ncRNAs involved in the ceRNA network; blue, ceRNA network; red, differentially expressed ncRNAs; intersection, a total of 369 differentially expressed ncRNAs involved in the ceRNA network were identified. (E) Venn diagram of the differentially expressed miRNAs involved in the ceRNA network; blue, miRNAs involved in the ceRNA network; red, differentially expressed miRNAs; intersection, a total of 23 differentially expressed miRNAs involved in the ceRNA network were identified. (F) Venn diagram of the differentially expressed mRNAs that serve as miRNA targets; blue, miRNA‐targeted mRNAs; red, differentially expressed mRNAs; intersection, a total of 117 differentially expressed mRNAs that serve as miRNA targets were identified. (G) ceRNA network of the differentially expressed ncRNAs, miRNAs and mRNAs in glioma; rectangle, ncRNA; ellipse, miRNA; diamond, mRNA; blue, downregulated RNAs; red, upregulated RNAs

To determine the genes related to malignant glioma tumorigenesis and progression, gene expression was analysed in 698 malignant glioma samples (169 GBM samples and 529 LGG samples) and compared to that in five normal brain samples. Ultimately, 4094 DEGs were identified: 2126 DEncRNAs (non‐miRNA) (Figure 2A), 67 DEmiRNAs (Figure 2B), and 1901 DEmRNAs (Figure 2C). The DEncRNAs (non‐miRNAs) accounted for almost half of the DEGs, which also highlights their importance in malignant glioma tumorigenesis and progression.

#### 3.1.2 ncRNA‐associated ceRNA networks among DEGs in malignant glioma

Considering that the ceRNA mechanism is one of the most common and critical mechanisms of ncRNAs, where an ncRNA competitively binds to a miRNA to modulate a regulatory complex and thus indirectly regulate mRNA expression,^42^, ^43^ DEGs were screened through the ceRNA network. First, the 2126 DEncRNAs (non‐miRNAs) were mapped to the ncRNA‐miRNA network in the miRcode database, and 369 ncRNA‐ 249 miRNA pairs remained (Figure 2D). Second, the 67 DEmiRNAs intersected with the 249 miRNAs that existed in the 369 ncRNA‐ 249 miRNA pairs and 23 miRNAs remained (Figure 2E). Third, the target mRNAs were identified through the miRDB database, miRTarBase database, and TargetScan database based on the remaining 23 miRNAs. Only the mRNAs that were recognized by all three databases were considered candidate target mRNAs. Finally, the target mRNAs were intersected with the DEmRNAs, and 117 mRNAs remained (Figure 2F). The ncRNA‐miRNA‐mRNA network was constructed based on these 509 genes (369 ncRNAs, 23 miRNAs and 117 mRNAs) (Figure 2G).

### 3.2 Potential genes for diagnosis, prognosis and therapy in malignant glioma

#### 3.2.1 Construction of the Cox and Lasso regression models

**FIGURE 3:**
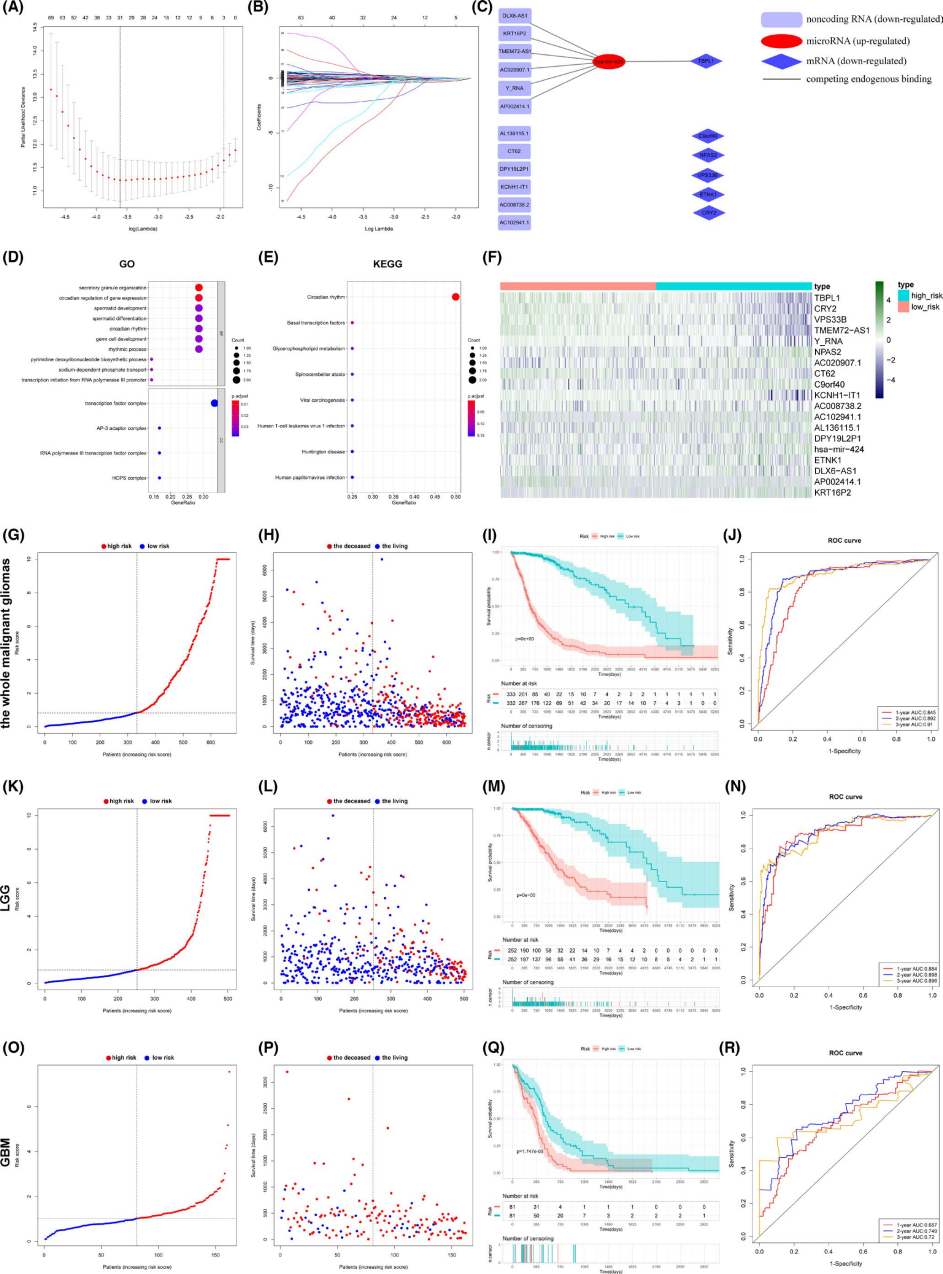
Critical genes for diagnosis, prognosis and therapy in glioma. (A) Cross‐validation fitting into Lasso regression to select the best model. For each λ value, around the mean of the target parameter shown in the red dot, a confidence interval for the target parameter can be obtained. The two dashed lines indicate two particular λ values: λ.min (dashed line on the left) refers to the minimum target parameter mean value in all λ values, while λ.1se (dashed line on the right) refers to the value of λ that gives the simplest model in a range of variances. (B) Tracing independent variable coefficients. Each curve represents the change in the trajectory of each independent variable coefficient. The ordinate is the coefficient value, and the lower abscissa is log (λ), while the upper abscissa is the number of nonzero coefficients in the model at this time. (C) Multivariate Cox regression yielded 19 genes to construct a model to predict and evaluate malignant glioma. Rectangle, ncRNA; ellipse, miRNAs; diamond, mRNAs; red, upregulated RNAs; blue, downregulated RNAs. (D) the risk group and the gene expression level of the 19 genes. (E) GO functional enrichment analysis: BP, biological process; CC, cell component. (F) KEGG functional enrichment analysis. The group of (G) the whole malignant glioma patients, (K) the LGG patients and (O) the GBM patients. Each dot represents a patient; red dot, high‐risk group patients; blue, low‐risk group patients. Survival state of (H) the whole glioma patients, (L) the LGG patients, and P) the GBM patients; Each dot represents a patient; The dots on the left of the dashed line, low‐risk group patients; the dots on the right of the dashed line, high‐risk group patients; Red dots, deceased patients; blue dots, living patients. Kaplan–Meier survival analysis of I) the whole glioma patients, (M) the LGG patients, and (Q) the GBM patients; red, patients in the high‐risk group; blue, patients in the low‐risk group; *p*<10^−20^ is shown as 0. TDROC curve of (J) the whole glioma patients, (N) the LGG patients, and (R) the GBM patients; red, 1‐year curve; blue, 2‐year curve; orange, 3‐year curve

The 509 genes in the ceRNA network are obviously not suitable for evaluating malignant glioma patients and illustrate the signalling pathways in malignant glioma because of their quantity. Therefore, we needed to further screen the most valuable genes among these 509 genes. First, 509 genes (369 ncRNAs, 23 miRNAs and 117 mRNAs) in the ncRNA‐miRNA‐mRNA network (Figure 2G) were incorporated in the univariate Cox regression model, and ultimately, 116 genes (51 ncRNAs, 2 miRNAs and 63 mRNAs) remained. Second, 116 genes were incorporated in the Lasso regression model, and the results showed that when logλ was close to −3.6, the deviance was the lowest; thus, 31 genes remained (19 ncRNAs, 1 miRNA and 11 mRNAs) (Figure 3A,B). Subsequently, the 31 genes were incorporated in the multivariate Cox regression model, and 19 genes remained: 12 ncRNAs (AC020907.1, Y_RNA, TMEM72‐AS1, KRT16P2, DLX6‐AS1, AP002414.1, AC008738.2, AC102941.1, AL136115.1, CT62, DPY19L2P1 and KCNH1‐IT1), where Y_RNA was a miscRNA, KRT16P2 and DPY19L2P1 were transcribed unprocessed pseudogenes, AP002414.1 was a processed pseudogene, and the others were lncRNAs—1 miRNA (hsa‐miR‐424) and 6 coding genes (TBPL1, C9ORF40, CRY2, ETNK1, NPAS2 and VPS33B) (Figure 3C). These results demonstrate the importance of the ncRNA‐associated network, not limited to the lncRNA‐associated network. Among these 19 genes, hsa‐miR‐424 was upregulated in malignant glioma, and the others were downregulated. Moreover, DLX6‐AS1, KRT16P2, TMEM72‐AS1, AC020907.1, Y_RNA and AP002414.1 would competitively bind to hsa‐miR‐424, while hsa‐miR‐424 would bind to TBPL1 mRNA according to the ceRNA network (Figure 3C). Functional enrichment analyses via the GO and KEGG databases were performed to identify the biological functions of the 19 genes (Figure 3D,E). The results showed that the circadian rhythm and secretory granule organization would be the most important biological processes.

#### 3.2.2 Evaluation of the model in malignant glioma

The model was validated at the internal and external levels. According to the expressions of these 19 genes (Figure 3F) which were used to calculate the median of risk score, the whole malignant glioma patients (Figure 3G), LGG patients (Figure 3K), and GBM patients (Figure 3O) were grouped into two categories, a high‐risk group and a low‐risk group. The numbers of deceased patients among the whole malignant glioma patients (Figure 3H), LGG patients (Figure 3L), and GBM patients (Figure 3P) in the high‐risk group were greater than that in the low‐risk group. Kaplan–Meier survival analysis showed that the survival time of the whole malignant glioma patients (Figure 3I), LGG patients (Figure 3M), and GBM patients (Figure 3Q) in the high‐risk group was significantly shorter than that in the low‐risk group (*p* < 10^−20^ in the whole malignant glioma group and LGG group, *p* = 1.747×10^−5^ in GBM group). A time‐dependent receiver operating characteristic (TDROC) curve was generated, and the area under the curve (AUC) of the whole malignant glioma patients was approximately 0.9 (Figure 3J), while the AUC of LGG patients was approximately 0.9 (Figure 3N), and the AUC of GBM patients was approximately 0.74 (Figure 3R). The concordance index (C‐index) of the whole malignant glioma patients was 0.836, with a standard error of 0.012, a lower value of 0.814, a higher value of 0.859, and a *p*‐value of 3.89 × 10^−186^. The C‐index of LGG patients was 0.859, with a standard error of 0.016, a lower value of 0.827, a higher value of 0.891, and a *p*‐value of 2.46 × 10^−106^. The C‐index of GBM patients was 0.643, with a standard error of 0.026, a lower value of 0.591, a higher value of 0.694, and a *p*‐value of 4.45 × 10^−8^.

Subsequently, external patients from the CGGA database were used to validate the model, and the results were similar. The whole malignant glioma patients (Figure S1A), LGG patients (Figure S1B), and GBM patients (Figure S1C) from the CGGA database were grouped into two categories, a high‐risk group and a low‐risk group, according to the risk score, which is based on the expression of these 19 genes. The numbers of deceased patients among the whole malignant glioma patients (Figure S1D), LGG patients (Figure S1E), and GBM patients (Figure S1F) in the high‐risk group were greater than that in the low‐risk group. Kaplan–Meier survival analysis showed that the number of deceased patients among the whole malignant glioma patients (Figure S1G), LGG patients (Figure S1H), and GBM patients (Figure S1I) in the high‐risk group was greater than that in the low‐risk group, and the survival time of the patients in the high‐risk group was significantly shorter than that in the low‐risk group (*p* < 10^−20^ in the whole malignant glioma group and LGG group, *p* = 2.119 × 10^−2^ in GBM group). The TDROC curve was generated, and the AUC of the whole malignant glioma patients was approximately 0.83 (Figure S1J), while the AUC of LGG patients was approximately 0.83 (Figure S1K), and the AUC of GBM patients was approximately 0.62 (Figure S1L). The C‐index of the whole malignant glioma patients was 0.741, with a standard error of 0.009, a lower value of 0.722, a higher value of 0.759, and a *p*‐value of 6.23 × 10^−147^. The C‐index of LGG patients was 0.759 with a standard error of 0.013, a lower value of 0.734, a higher value of 0.785, and a *p*‐value of 3.87 × 10^−86^. The C‐index of GBM patients was 0.565, with a standard error of 0.018, a lower value of 0.529, a higher value of 0.601, and a *p*‐value of 4.23 × 10^−4^.

### 3.3 Biological significance of the model in malignant glioma

#### 3.3.1 SNV analysis in malignant glioma

**FIGURE 4:**
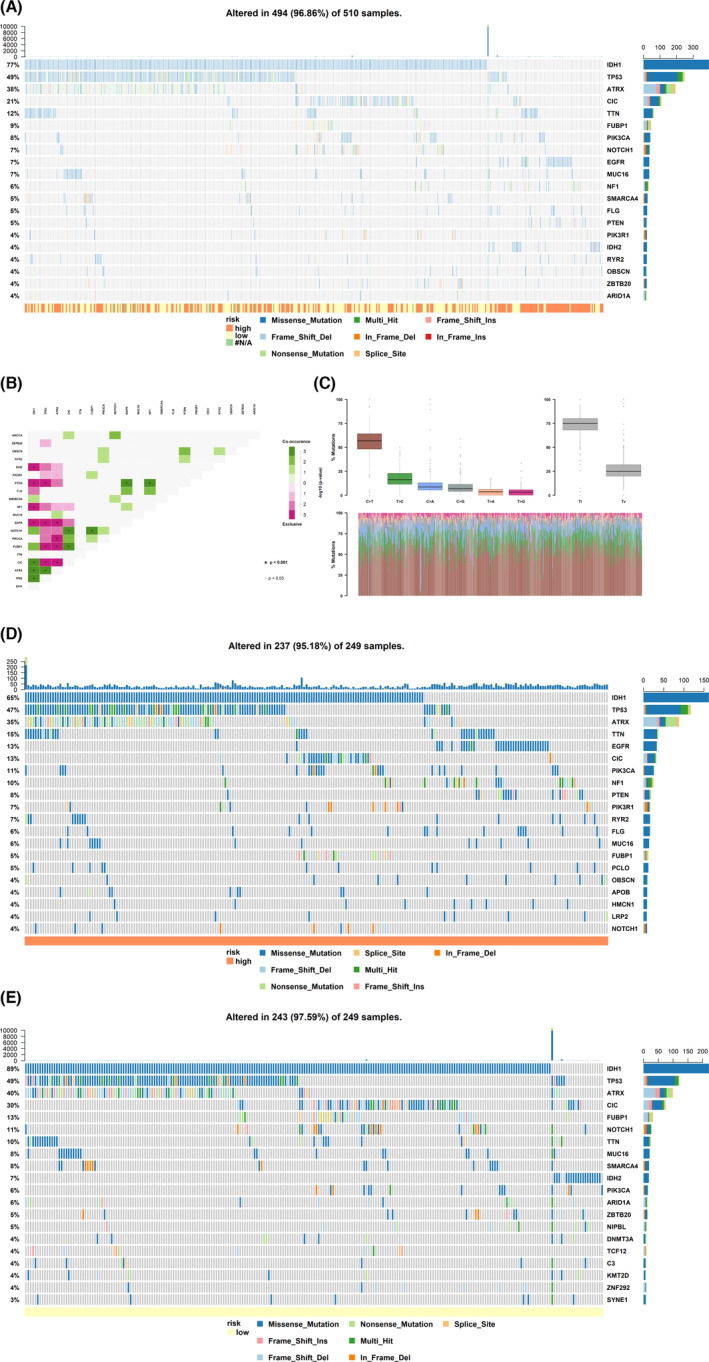
SNV analysis. (A) SNV signatures in a total of 510 malignant glioma patients. (B) Correlations between the top 20 mutated genes. (C) Types and proportions of mutations. Ti, transition; Tv, transversion; A, adenine; T, thymine; C, cytosine; G, guanine. (D) SNV signatures in patients in the high‐risk group based on the model. (E) SNV signatures in patients in the low‐risk group based on the model

Before we could further determine and illustrate the molecular mechanisms among these genes, we must first examine the mutations in these 19 genes in glioma because if there were mutations in these 19 genes, the sequences and structures of their transcripts and proteins would change, thus hampering further analysis based on the transcripts and structures. The 510 malignant glioma samples were examined, and the top 20 mutated genes in malignant glioma were IDH1, TP53, ATRX, CIC, TTN, FUBP1, PIK3CA, NOTCH1, EGFR, MUC16, NF1, SMARCA4, FLG, PTEN, PIK3R1, IDH2, RYR2, OBSCN, ZBTB20 and ARID1A, where the most common type of mutation was missense (Figure 4A). The relations among these top 20 mutated genes were also examined (Figure 4B); for example, IDH1 mutations usually co‐occurred with CIC, ARTX and TP53 mutations and exclusively with IDH2, PTEN, NF1 and EGFR mutations. Transition (Ti) was the major mutation type (mainly cytosine transitions to thymine or thymine transitions to cytosine), which accounted for approximately 75% of mutations, and transversion (Tv) accounted for approximately 25% of mutations (Figure 4C). Fortunately, mutations in the 19 genes were not found, suggesting that the effectiveness of the model would not be affected by gene mutations in malignant glioma.

Furthermore, the samples were grouped into two categories, a high‐risk group and a low‐risk group, based on the expression of the 19 genes to examine the incidence rate of mutations in various genes (Figure 4D,E). IDH1, TP53 and ATRX were the top 3 mutated genes in both the high‐risk group and the low‐risk group. The incidence rate of IDH1 mutation in the low‐risk group (89%) was much higher than that in the high‐risk group (65%), and simultaneously, the incidence rate of ATRX mutation in the low‐risk group (40%) was much higher than that in the high‐risk group (35%), while the incidence rate of TP53 mutation in the low‐risk group (49%) was not significantly different from that in the high‐risk group (47%). These phenomena are coincident with the consensus that mutations in IDH1 and ATRX are the initiating events for the development of many gliomas, and their presence dictates favourable clinical behaviour.^44^, ^45^, ^46^ This result confirms that our model based on the 19 genes would be reliable. Furthermore, as we know, IDH is the critical metabolic enzyme that catalyses the oxidative decarboxylation of isocitrate to α‐ketoglutarate (αKG), NAD(P)H and CO_2_, while mutant enzymes produce (R)‐2‐hydroxyglutarate, which in turn inhibits αKG‐dependent dioxygenase function, resulting in a global hypermethylation phenotype.^47^ DNA methylation is one of the most important processes in gene expression regulation and usually suppresses gene expression. Interestingly, among these 19 genes, 18 were downregulated. Therefore, we conjecture that this hypermethylation may influence the expression of these 19 genes.

#### 3.3.2 Circadian rhythm in malignant glioma

(This section includes only the declarative results. Should you read this manuscript for the first time, we strongly suggest you read Section 4.1 at first.) Functional enrichment analyses via the GO and KEGG databases were performed to identify the biological functions of the 19 genes (Figure 3D,E). The results showed that the circadian rhythm and secretory granule organization would be the most important biological processes.

**FIGURE 5:**
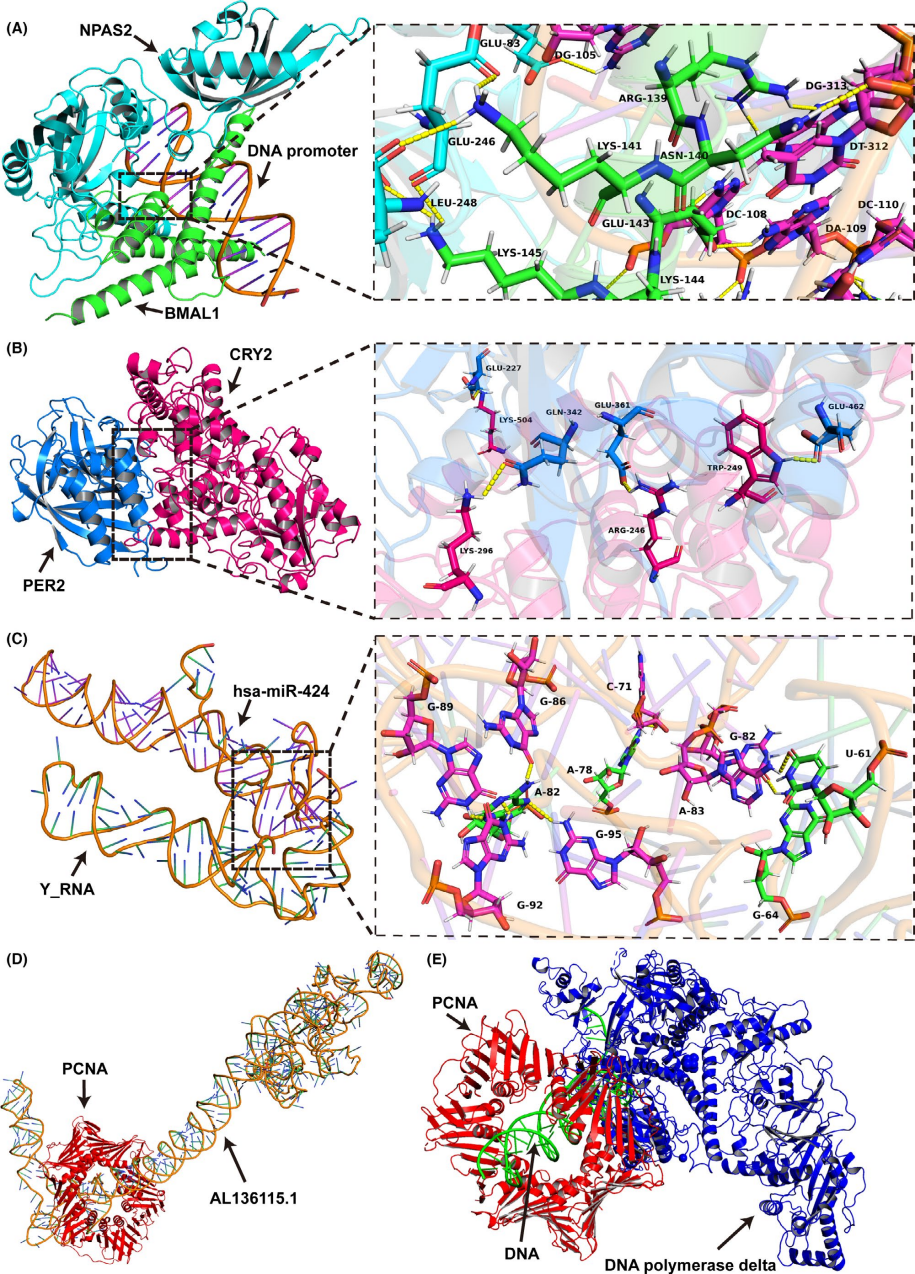
Molecular docking. (A) The interactions among NPAS2, BMAL1, and DNA promoters. (B) The interaction between CRY2 and PER2. (C) The interaction between hsa‐miR‐424 and Y_RNA. (D) The interaction between the PCNA trimer and AL136115.1. AL136115.1 competitively passes through the cavity of PCNA, which hinders the DNA from going through the same site. (E) The interactions among PCNA, DNA, and Pol δ. The PCNA trimer forms a hexagon‐like structure with a cavity where DNA passes through PCNA and binds to Pol δ to replicate the lagging strandy.^66^

CRY2 and NPAS2 are the core components of the circadian clock.^48^, ^49^ Moreover, NPAS2 interacts with BMAL1, and the compound then binds to the E‐box of the CRY2 and PER2 promoters to facilitate expression.^50^ Furthermore, CRY2 heterodimerizes with PER2 to bind to the transcription factors NR4A2, HNF4A, PPARA and NR1D1 to facilitate their activation.^50^ Therefore, can TBPL1, a transcription factor,^51^, ^52^ bind to the NPAS2 promoter to facilitate its expression, thus promoting CRY2 expression and further activating NR4A2, HNF4A, PPARA and NR1D1? To test whether TBPL1 could bind to the NPAS2 promoter to facilitate its expression, transcription factor binding site predictions were performed, and the results showed that the NPAS2 promoter has four potential binding sites, with the possibility of more than 0.9 to which TBPL1 would bind (Table S2). To test whether NPAS2 could interact with BMAL1 and the DNA promoter, molecular docking analysis was performed, and the results revealed that NPAS2 would interact with BMAL1 and bind to the E‐box of the DNA promoter (Figure 5A), thus promoting PER2 and CRY2 expression. Moreover, CRY2 heterodimerizes with PER2 (Figure 5B) to bind to the transcription factors NR4A2, HNF4A, PPARA and NR1D1 to facilitate their activation.

As 6 coding genes (TBPL1, C9ORF40, CRY2, ETNK1, NPAS2 and VPS33B) were identified, we asked whether NR4A2, HNF4A, PPARA and NR1D1, as transcription factors, promote or suppress the expression of the other three genes (C9ORF40, ETNK1, or VPS33B). To examine whether NR4A2, HNF4A, PPARA and NR1D1 could promote or suppress the expression of C9ORF40, ETNK1, or VPS33B, similar transcription factor binding site predictions were performed, and the results showed that only the transcription factor NR4A2 would bind to the promoters of C9ORF40, ETNK1 and VPS33B to promote or suppress their expression (Table S2).

#### 3.3.3 Tumour immune microenvironment in malignant glioma

**FIGURE 6:**
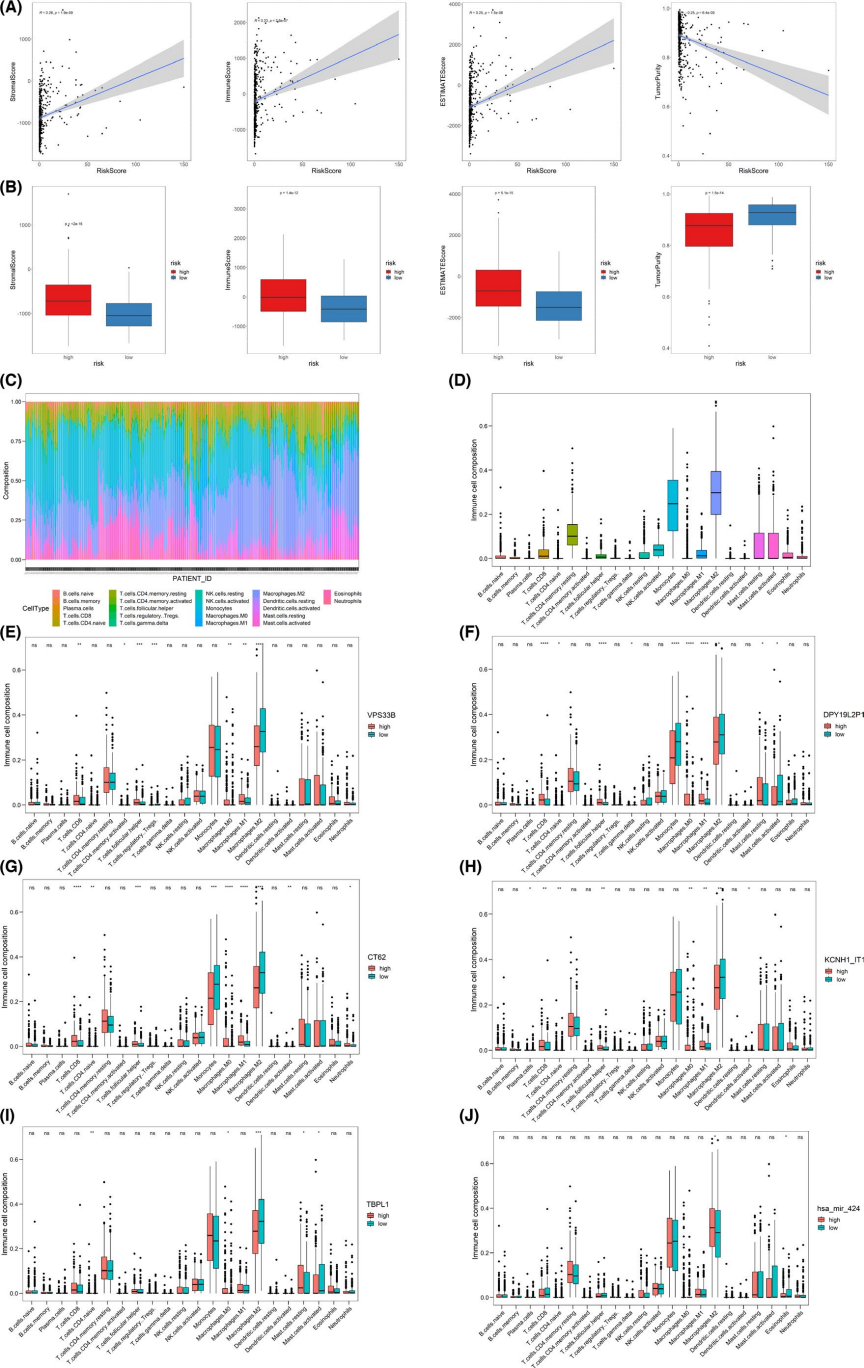
Immune infiltration analysis. A) Linear correlations between the risk score and stromal score, immune score, ESTIMATE score, and tumour purity. ESTIMATE, Estimation of Stromal and Immune Cells in Malignant Tumour Tissues using Expression Data. B) Boxplot of the correlations among the risk score, stromal score, immune score, ESTIMATE score, and tumour purity. C) Landscape of infiltrating immune cells. D) Relative proportions of infiltrating immune cells. E) Relative proportions of infiltrating immune cells based on the expression of VPS33B (high or low). F) Relative proportions of infiltrating immune cells based on the expression of DPY19L2P1 (high or low). G) Relative proportions of infiltrating immune cells based on the expression of CT62 (high or low). H) Relative proportions of infiltrating immune cells based on the expression of KCNH1‐IT1 (high or low). I) Relative proportions of infiltrating immune cells based on the expression of TBPL162 (high or low). J) Relative proportions of infiltrating immune cells based on the expression of hsa‐mir‐424 (high or low). *, *p* < 0.05; **, *p* < 0.01; ***, *p* < 0.005; ****, *p* < 0.001; ns, not significant

(This section includes only the declarative results. Should you read this manuscript for the first time, we strongly suggest you read Sections 4.1 and 4.2 at first.) Accumulating evidence indicates that VPS33B plays a critical role in vesicle‐mediated transport and organization.^53^, ^54^, ^55^, ^56^ It has been reported that the ncRNAs CT62, DPY19L2P1 and KCNH1‐IT1 can be delivered by exosomes, while AC008738.2, AC102941.1 and AL136115.1 have not been identified.^57^, ^58^ Therefore, VPS33B would promote exosomes carrying CT62, DPY19L2P1 and KCNH1‐IT1 to other cells, thus regulating the tumor microenvironment. Considering that immune cells are an important component of the tumor environment, glioma‐derived exosomes would deliver CT62, DPY19L2P1 and KCNH1‐IT1 to immune cells, regulating the tumour immune microenvironment.^59^, ^60^ To test whether CT62, DPY19L2P1 and KCNH1‐IT1 could regulate the tumour immune microenvironment, immune analyses were performed. The results revealed that as the risk score increased, the proportion of stromal cells and immune cells increased while tumour purity decreased (Figure 6A,B), indicating that immune cells play a critical role in malignant glioma tumorigenesis and progression. Moreover, M2 macrophages were the most abundant immune cells in the malignant glioma microenvironment (Figure 6C,D), suggesting that M2 macrophages would play an important role in malignant glioma tumorigenesis and progression. The proportions of M2 macrophages in the VPS33B (Figure 6E), DPY19L2P1 (Figure 6F), CT62 (Figure 6G) and KCNH1‐IT1 (Figure 6H) low expression groups were higher than those in the high expression groups, while the proportions of M0 macrophages and M1 macrophages were lower than those in the high expression groups. These results suggest that VPS33B, CT62, DPY19L2P1 and KCNH1‐IT1 would suppress M0 macrophage and M1 macrophage differentiation into M2 macrophages. What's more, considering TBPL1 and hsa‐mir‐424 that locate upstream of the pathways, the effects of TBPL1 and hsa‐mir‐424 have also been validated. The results show that the proportion of M2 macrophages in the TBPL1 (Figure 6I) low expression groups was higher than that in the high expression groups, while the proportion of M2 macrophages in the hsa‐mir‐424 (Figure 6J) low expression groups was lower than that in the high expression groups. This, again, verifies that our model is reliable.

#### 3.3.4 Cellular senescence in malignant glioma

(This section includes only the declarative results. Should you read this manuscript for the first time, we strongly suggest you read Sections 4.1, 4.2, 4.3 at first.) We have illustrated that Y_RNA, AC020907.1, TMEM72‐AS1, KRT16P2, DLX6‐AS1 and AP002414.1 would competitively bind to hsa‐miR‐424 and that hsa‐miR‐424 could bind to TBPL1 mRNA. To further verify these findings, molecular docking was performed. The results revealed that Y_RNA (Figure 5C), AC020907.1 (Figure S2A), TMEM72‐AS1 (Figure S2B), KRT16P2 (Figure S2C), DLX6‐AS1 (Figure S2D) and AP002414.1 (Figure S2E) could competitively bind to hsa‐miR‐424, while hsa‐miR‐424 could bind to TBPL1 mRNA (Figure S2F).

Although C9ORF40 has no correlation with either the circadian rhythm or secretory granule organization, it plays a critical role in cellular senescence by activating the p21 pathway.^61^ ^61^ C9ORF40 activates p21 and thus inhibits proliferation by the CDK or proliferating cell nuclear antigen (PCNA) signalling pathway and simultaneously promotes apoptosis by the caspase‐3 signalling pathway.^62^, ^63^, ^64^, ^65^ To test whether the other three ncRNAs, AC008738.2, AC102941.1, and AL136115.1, could bind to p21, CDK, PCNA, or caspase‐3, molecular docking analysis was performed. The results revealed that only PCNA would interact with the ncRNAs AL136115.1 (Figure 5D), AC008738.2 (Figure S2G), and AC102941.1 (Figure S2H). In eukaryotes, the PCNA trimer forms a hexagon‐like structure with a cavity where DNA passes through PCNA and binds to DNA polymerase δ (Pol δ) to replicate the lagging strand (Figure 5E) and cooperates with flap endonuclease 1 (FEN1) to process the Okazaki fragments for ligation.^66^However, the DNA binding cavity for PCNA would be occupied by the ncRNAs AC008738.2, AC102941.1, and AL136115.1, hindering and impeding DNA replication and thus suppressing proliferation and other cell cycle processes in malignant glioma.

### 3.4 Clinical significance of the circadian rhythm, tumour immune microenvironment, and cellular senescence pathways in malignant glioma

The eleven genes within the circadian rhythm pathway (AC020907.1, Y_RNA, TMEM72‐AS1, KRT16P2, DLX6‐AS1, AP002414.1, hsa‐miR‐424, TBPL1, NPAS2, CRY2, and ETNK1) were used to construct a model to evaluate the importance of these genes in malignant glioma. The patients were grouped into two groups based on the expression of these genes: a high‐risk group and a low‐risk group (Figure S3A). Kaplan–Meier survival analysis showed that the number of deceased patients in the high‐risk group was greater than that in the low‐risk group, and the survival time of the patients in the high‐risk group was significantly shorter than that in the low‐risk group (*p* < 10^−20^) (Figure S3B,C). The TDROC curve was generated, and the AUC was approximately 0.9, with a 1‐year AUC of 0.866, a 2‐year AUC of 0.889, and a 3‐year AUC of 0.887 (Figure S3D). The C‐index was 0.841, with a standard error of 0.017, a lower value of 0.806, a higher value of 0.876, and a *p*‐value of 2.60 × 10^−82^. These results reveal that this model would also be accurate, highlighting that the circadian rhythm pathway and its related genes would be critical for malignant glioma and its evaluation.

Fourteen genes within the cellular senescence pathway (AC020907.1, Y_RNA, TMEM72‐AS1, KRT16P2, DLX6‐AS1, AP002414.1, hsa‐miR‐424, TBPL1, NPAS2, CRY2, C9ORF40, AL136115.1, AC102941.1, and AC008738.2) were used to construct a model to evaluate the importance of these genes in malignant glioma. The patients were grouped into two groups based on the expression of these genes: a high‐risk group and a low‐risk group (Figure S3E). Kaplan–Meier survival analysis showed that the number of deceased patients in the high‐risk group was greater than that in the low‐risk group, and the survival time of the patients in the high‐risk group was significantly shorter than that in the low‐risk group (*p* < 10^−20^) (Figure S3F,G). The TDROC curve was generated, with a 1‐year AUC of 0.866, a 2‐year AUC of 0.900, and a 3‐year AUC of 0.906 (Figure S3H). The C‐index was 0.849, with a standard error of 0.016, a lower value of 0.817, a higher value of 0.881, and a *p*‐value of 4.18 × 10^−103^. Overall, this model would also be accurate, highlighting that the cellular senescence pathway and its related genes would be critical for malignant glioma and its evaluation.

Fourteen genes within the tumour immune microenvironment pathway (AC020907.1, Y_RNA, TMEM72‐AS1, KRT16P2, DLX6‐AS1, AP002414.1, hsa‐miR‐424, TBPL1, NPAS2, CRY2, VPS33B, CT62, DPY19L2P1, and KCNH1‐IT1) were used to construct a model to evaluate the importance of these genes in malignant glioma. The patients were grouped into two groups based on the expression of these genes: a high‐risk group and a low‐risk group Figure S3I). Kaplan–Meier survival analysis showed that the number of deceased patients in the high‐risk group was greater than that in the low‐risk group, and the survival time of the patients in the high‐risk group was significantly shorter than that in the low‐risk group (*p* < 10^−20^) (Figure S3J,K). The TDROC curve was generated, with a 1‐year AUC of 0.884, a 2‐year AUC of 0.888, and a 3‐year AUC of 0.878 Figure S3L). The C‐index was 0.845, with a standard error of 0.019, a lower value of 0.808, a higher value of 0.882, and a *p*‐value of 1.62 × 10^−73^. Overall, this model would also be accurate, highlighting that the tumour immune microenvironment pathway and its related genes would be critical for malignant glioma and its evaluation.

Since all three pathways act through AC020907.1, Y_RNA, TMEM72‐AS1, KRT16P2, DLX6‐AS1, AP002414.1, hsa‐miR‐424, TBPL1, NPAS2, and CRY2, these genes could serve as potential therapeutic targets for malignant glioma. Knockdown of hsa‐miR‐424 or overexpression of AC020907.1, Y_RNA, TMEM72‐AS1, KRT16P2, DLX6‐AS1, AP002414.1, and TBPL1 could regulate these three pathways to suppress malignant glioma tumorigenesis and progression.

### 3.5 Experiment validation

**FIGURE 7:**
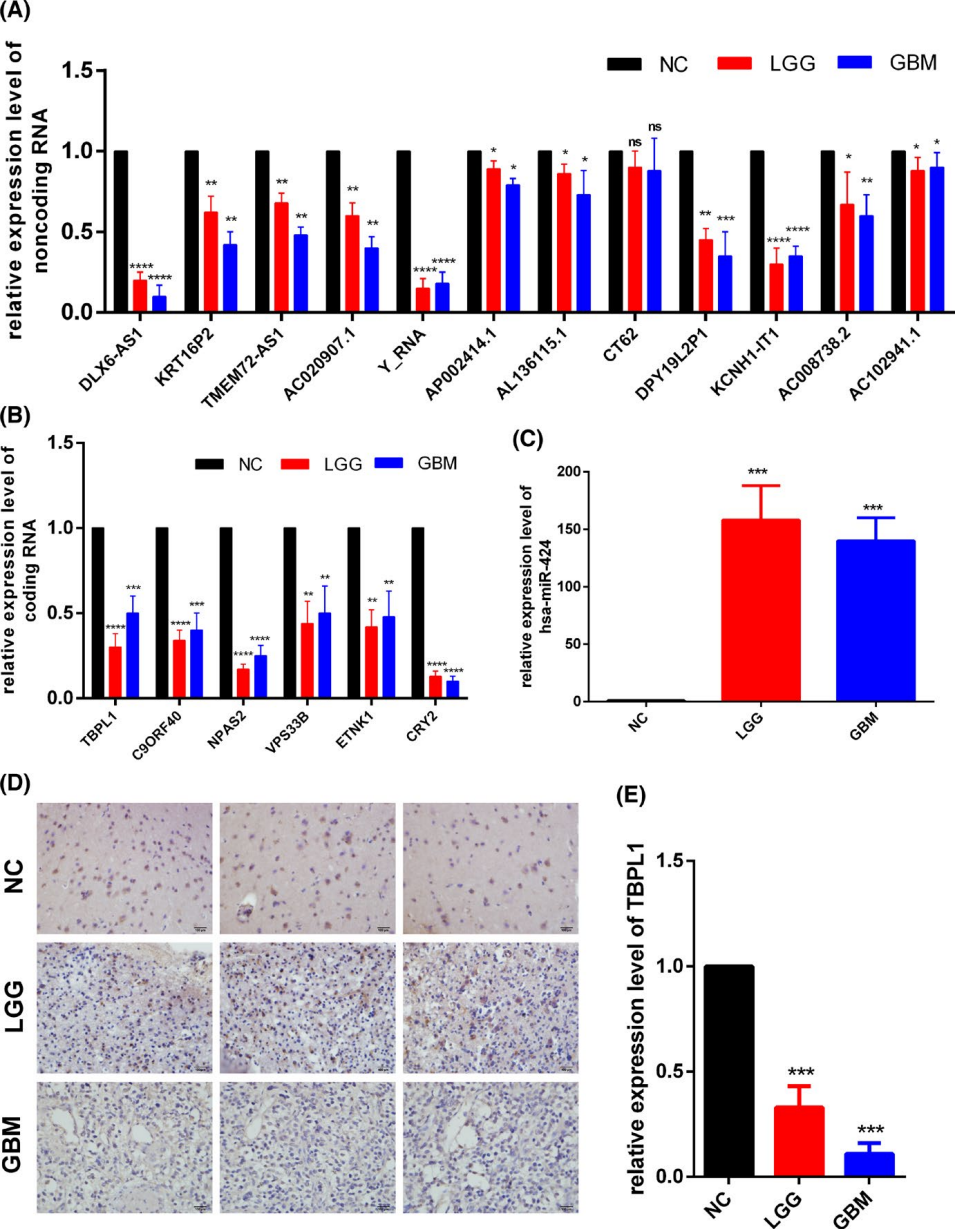
Experiment validation of the 19‐gene model and core protein TBPL1. Relative expression level of (A) ncRNA, (B) coding RNA and (C) microRNA tested by PCR. (D,E), Relative expression level of TBPL1 protein detected by IHC. *, *p* < 0.05; **, *p* < 0.01; ***, *p* < 0.005; ****, *p* < 0.001; ns, not significant

The PCR assays were performed to verify the 19‐gene model preliminarily (Figure 7A–C). Nearly all the genes were consistent with our conclusions except CT62. The ncRNAs (DLX6‐AS1, KRT16P2, TMEM72‐AS1, AC020907.1, Y_RNA, AP002414.1, AL136115.1, DPY19L2P1, KCNH1‐IT1, AC008738.2, and AC102941.1) and coding RNAs (TBPL1, C9ORF40, NPAS2, VPS33B, ETNK1, and CRY2) were low expressed in both LGG and GBM, while microRNA (hsa‐miR‐424) were high expressed in both LGG and GBM. In particular, DLX6‐AS1, Y_RNA, KCNH1‐IT1, hsa‐miR‐424, TBPL1, C9ORF40, NPAS2, and CRY2 have significance with *p* < 0.001, which indicates the priority of these genes in further experiments. Further, the IHC (Figure 7D,E) assays were performed to verify the expression of core protein TBPL1 which lies upstream of the three pathways. The results of IHC indicate that the expressions of TBPL1 in LGG and GBM were downregulated, and the expression of TBPL1 in GBM was much lower than LGG, which was consistent with our model.

## 4 DISCUSSION

### 4.1 Circadian rhythm pathway

After identifying DEGs, constructing ceRNA network, performing Cox/Lasso regression model, and evaluating the model, we obtained a novel 19‐gene model (Figure 3C), including 12 ncRNAs (AC020907.1, Y_RNA, TMEM72‐AS1, KRT16P2, DLX6‐AS1, AP002414.1, AC008738.2, AC102941.1, AL136115.1, CT62, DPY19L2P1 and KCNH1‐IT1), where Y_RNA is a miscRNA, KRT16P2 and DPY19L2P1 are transcribed unprocessed pseudogenes, AP002414.1 is a processed pseudogene, and the others are lncRNAs—1 miRNA (hsa‐miR‐424), and 6 coding genes (TBPL1, C9ORF40, CRY2, ETNK1, NPAS2, and VPS33B). But what's the biological function of these genes in malignant glioma? As mutations in the 19 genes were not found in malignant glioma (Figure 4), we could further determine the correlations between the 19 genes and malignant glioma. Functional enrichment analyses via the GO and KEGG databases were performed to identify the biological functions of the 19 genes (Figure 3D,E). The results showed that the circadian rhythm and secretory granule organization were the most important biological processes. First, we will discuss the circadian rhythm.

We previously found some relationships among these 19 genes—Y_RNA (Figure 5C), AC020907.1 (Figure S2A), TMEM72‐AS1 (Figure S2B), KRT16P2 (Figure S2C), DLX6‐AS1 (Figure S2D), and AP002414.1 (Figure S2E)—which could competitively bind to hsa‐miR‐424, and hsa‐miR‐424, which could bind to TBPL1 mRNA (Figure S2F). These correlations, however, are not enough to explain the biological functions of the 19 genes in malignant glioma tumorigenesis and progression. We observed that among the 6 coding genes (TBPL1, C9ORF40, CRY2, ETNK1, NPAS2, and VPS33B) (Figure 3C), CRY2 and NPAS2 were the core components of the circadian clock.^48^, ^49^ Moreover, NPAS2 interacts with BMAL1, and the compound then binds to the E‐box of the CRY2 and PER2 promoters (Figure 5A) to facilitate their expression.^50^ Furthermore, CRY2 heterodimerizes with PER2 (Figure 5B) to bind to the transcription factors NR4A2, HNF4A, PPARA, and NR1D1 to facilitate their activation.^50^ Therefore, we asked whether TBPL1, a transcription factor,^51^, ^52^ can bind to the NPAS2 promoter to facilitate its expression, thus promoting CRY2 expression and further activating NR4A2, HNF4A, PPARA, and NR1D1. Transcription factor binding site predictions showed that TBPL1 would bind to the NPAS2 promoter (Table S2), indicating that TBPL1 would promote activation of NR4A2, HNF4A, PPARA, and NR1D1 indirectly through NPAS2/CRY2. Further, can NR4A2, HNF4A, PPARA, and NR1D1, as transcription factors, promote or suppress the expression of C9ORF40, ETNK1, or VPS33B? Similar transcription factor binding site predictions showed that only the transcription factor NR4A2 would bind to the promoters of C9ORF40, ETNK1, and VPS33B to promote or suppress their expression (Table S2). Moreover, VPS33B expression is regulated by the circadian rhythm,^67^ which is consistent with our conclusion. Furthermore, ethanolamine kinase 1 (ETNK1) is a rate‐controlling enzyme in phosphatidylethanolamine biosynthesis, and its low expression would promote the proliferation and invasion of gastric cancer, larynx‐ or tongue‐derived squamous cell carcinoma, among others,^68^, ^69^, ^70^ indicating that TBPL1 would promote glioma tumorigenesis via the NPAS2/CRY2/NR4A2/ETNK1 pathway. Moreover, accumulating evidence indicates that PER2 plays important and critical roles in tumour suppression and the DNA damage response through the NPAS2/BMAL1 complex, which regulates TP53 and C‐MYC.^71^, ^72^Overall, the noncoding RNAs AC020907.1, Y_RNA, TMEM72‐AS1, KRT16P2, DLX‐AS1, and AP002414.1 competitively bind to miR‐424 to promote TBPL1 expression, thus promoting NPAS2 expression. Furthermore, CRY2 and PER2 would suppress glioma tumorigenesis by suppressing ETNK1 expression through the transcription factor NR4A2 or by suppressing C‐MYC or TP53 expression through the NPAS2‐BMAL1 complex (Figure 1B).

Interestingly, Ben Franklin's aphorism ‘early to bed, early to rise, makes a man healthy, wealthy, and wise’ also suggests the potential importance of the circadian rhythm in health. Regardless of bacteria or eukaryotes, circadian rhythms play an important role in controlling a variety of physiological processes, and disruptions to normal circadian biology can cause many diseases.^73^ Many physiological processes, including hormone secretion, drug and xenobiotic metabolism, glucose homeostasis, cell cycle progression, and tumorigenesis, are regulated by the circadian rhythm,^49^, ^74^, ^75^. Accumulating evidence indicates that circadian rhythm disorders cause many diseases.^76^, ^77^, ^78^, ^79^, ^80^, ^81^ Therefore, understanding the correlation between the circadian rhythm and disease is critical and important for understanding the underlying mechanisms and potential therapies. Our work reveals that glioma tumorigenesis is significantly correlated with the circadian rhythm. We strongly suggest not staying up late if you can go to bed early.

### 4.2 Tumour immune microenvironment

During the process of illustrating the circadian rhythm pathway, among the 6 coding genes, we exhibited the biological functions of TBPL1, NPAS2, CRY2, and ETNK1; therefore, what are the roles of VPS33B and C9ORF40 in malignant glioma? As GO and KEGG functional enrichment analyses revealed that these genes are also related to secretory granule organization (Figure 3D,E), we are going to discuss secretory granule organization.

Accumulating evidence indicates that VPS33B plays a critical role in vesicle‐mediated transport and organization.^53^, ^54^, ^55^, ^56^ Therefore, given that exosomes, a kind of vesicle that mediates communication between cells by transferring ncRNAs, promote tumorigenesis,^82^, ^83^, ^84^ can VPS33B promote exosomes carrying ncRNAs to other cells in addition to malignant glioma and thus regulate the tumor microenvironment? First, the following 6 ncRNAs have not been discussed until now: AC008738.2, AC102941.1, AL136115.1, CT62, DPY19L2P1, and KCNH1‐IT1. Therefore, do exosomes carry these 6 ncRNAs? Next, do these 6 ncRNAs regulate the tumour microenvironment? It has been reported that the ncRNAs CT62, DPY19L2P1, and KCNH1‐IT1 can be delivered by exosomes, while AC008738.2, AC102941.1, and AL136115.1 have not been identified.^57^, ^58^ Therefore, VPS33B would promote exosomes carrying CT62, DPY19L2P1, and KCNH1‐IT1 to other cells, thus regulating the tumour microenvironment. Considering that immune cells are an important component of the tumour environment, glioma‐derived exosomes would deliver CT62, DPY19L2P1, and KCNH1‐IT1 to immune cells, regulating the tumour immune microenvironment.^59^, ^60^ Immune analyses revealed that as the risk score increased, the proportions of stromal cells and immune cells increased, while tumour purity decreased (Figure 6A,B), indicating that immune cells play a critical role in malignant glioma tumorigenesis and progression. We noticed that M2 macrophages were the most abundant cell type in the malignant glioma microenvironment (Figure 6C,D), suggesting that M2 macrophages would play an important role in malignant glioma tumorigenesis and progression. Moreover, the proportions of M2 macrophages in the VPS33B (Figure 6E), DPY19L2P1 (Figure 6F), CT62 (Figure 6G), and KCNH1‐IT1 (Figure 6H) low expression groups were higher than those in the high expression groups, while the proportions of M0 macrophages and M1 macrophages in the low expression groups were lower than those in the high expression groups. These results suggest that VPS33B, CT62, DPY19L2P1, and KCNH1‐IT1 would suppress M0 macrophage and M1 macrophage progression into M2 macrophages. Given that VPS33B plays a critical role in vesicle‐mediated transport and organization^53^, ^54^, ^55^, ^56^ and that the ncRNAs CT62, DPY19L2P1, and KCNH1‐IT1 can be delivered by exosomes,^57^, ^58^ VPS33B would promote the organization and release of exosomes that carry CT62, DPY19L2P1 and KCNH1‐IT1 to immune cells, suppressing M0 macrophage and M1 macrophage progression into M2 macrophages. What's more, considering TBPL1 and hsa‐mir‐424 that locate upstream of the pathways, the effects of TBPL1 and hsa‐mir‐424 have also been validated. The results show that the proportion of M2 macrophages in the TBPL1 (Figure 6I) low expression groups was higher than that in the high expression groups, while the proportion of M2 macrophages in the hsa‐mir‐424 (Figure 6J) low expression groups was lower than that in the high expression groups. This, again, verifies our model is reliable. In M2 macrophages, arginine metabolism converts arginine into ornithine and polyamines, promoting proliferation and repair through polyamine and collagen synthesis, fibrosis and other tissue remodelling functions, thus promoting tumorigenesis.^85^, ^86^, ^87^, ^88^Therefore, the low expression of CT62, DPY19L2P1, KCNH1‐IT1 and VPS33B in malignant glioma would promote the formation of M2 macrophages and thus promote malignant glioma tumorigenesis and progression (Figure 1B).

Interestingly, for a long time, the immune system was considered to play a protective role in tumour development.^89^. Accumulating evidence, however, indicates that various types of immune and inflammatory cells are frequently present within tumours and are strongly correlated with tumours.^90^Exosomes, vesicles of endocytic origin released by cells, mediate communication between cells by transferring mRNAs, ncRNAs, liquids and proteins, thus promoting cell processes such as antigen presentation, tumorigenesis, neurodegeneration, immune responses, and intracellular trafficking.^82^, ^83^, ^84^, ^91^, ^92^Overall, accumulating evidence highlights the important relationship between tumour cells and other cells mediated by exosomes. In our work, we reveal that the immune system plays an undeniably important role in the development of malignant glioma through exosomes. Thus, understanding the relationship among malignant glioma, the immune microenvironment, and exosomes is vital for illustrating the underlying mechanisms and improving malignant glioma therapy.

### 4.3 Cellular senescence

After illustrating the circadian rhythm pathway and tumour immune microenvironment pathway, only one coding gene, C9ORF40, and three ncRNAs, AC008738.2, AC102941.1 and AL136115.1, remain to be illustrated. Although C9ORF40 has no relationship with either the circadian rhythm or secretory granule organization, it plays a critical role in cellular senescence by activating the p21 pathway.^61^p21, which is encoded by CDKN1A, is a key protein in cellular senescence and causes G1 phase arrest by interacting with CDK1 or CDK2 to disrupt the interaction between substrates and CDKs, thus inhibiting cell cycle progression.^62^, ^63^, ^64^p21 can directly bind to PCNA and inhibit its activation, which inhibits DNA polymerase activity, transcription, and excision repair functions[65]. Moreover, p21 induces apoptosis by activating the caspase‐3‐mediated signalling pathway.^62^, ^63^Overall, C9ORF40 activates p21 and thus inhibits proliferation by the CDK or PCNA signalling pathway and simultaneously promotes apoptosis by the caspase‐3 signalling pathway.

What are the roles of the ncRNAs AC008738.2, AC102941.1 and AL136115.1 in the cellular senescence pathway? One possible explanation is that the ncRNAs AC008738.2, AC102941.1 and AL136115.1 would interact with the p21, CDK, PCNA and caspase‐3 proteins to regulate cellular senescence. Therefore, the question is whether p21, CDK, PCNA and caspase‐3 are RNA‐binding proteins. PCNA was identified as an RNA‐binding protein[93], while the others were not. To test whether the ncRNAs AC008738.2, AC102941.1 and AL136115.1 could bind to p21, CDK, PCNA and/or caspase‐3, molecular docking analysis was performed. The results revealed that only PCNA would interact with the ncRNAs AL136115.1 (Figure 5D), AC008738.2 (Figure S2G), and AC102941.1 (Figure S2H). In eukaryotes, the PCNA trimer forms a hexagon‐like structure with a cavity where DNA passes through PCNA and binds to DNA polymerase δ (Pol δ) to replicate the lagging strand (Figure 5E) and cooperates with flap endonuclease 1 (FEN1) to process the Okazaki fragments for ligation[66]. However, the DNA binding cavity for PCNA would be occupied by the ncRNAs AC008738.2, AC102941.1 and AL136115.1, hindering and impeding DNA replication and thus suppressing proliferation and other cell cycle processes in malignant glioma.

In particular, the process of cellular senescence is considered a state of irreversible growth arrest. Cancer cells, however, do not enter this state and begin to proliferate infinitely.^94^Cellular senescence was found to be mediated by the two main tumour suppressor pathways of the cell: the ARF/p53 and INK4a/RB pathways.^94^, ^95^, ^96^Overall, overwhelming evidence indicates that cellular senescence is a critical feature of mammalian cells to suppress tumorigenesis. In our work, we also revealed that cellular senescence is significantly correlated with glioma tumorigenesis (Figure 1B).

## 5 CONCLUSIONS

- Nineteen genes (12 ncRNAs: AC020907.1, Y_RNA, TMEM72‐AS1, KRT16P2, DLX6‐AS1, AC008738.2, AC102941.1, AL136115.1, CT62, DPY19L2P1 and KCNH1‐IT1; 1 miRNA: hsa‐miR‐424; and 6 coding genes: TBPL1, C9ORF40, CRY2, ETNK1, NPAS2 and VPS33B) were used to construct a model to evaluate malignant glioma patients (AUC of approximately 0.91 and C‐index of approximately 0.84). These 19 genes could be used as diagnostic/prognostic biomarkers for malignant glioma.
- The model is very suitable for LGG and is also suitable for GBM (AUC of approximately 0.90 and C‐index of approximately 0.86 in LGG and AUC of approximately 0.75 and C‐index of approximately 0.69 in GBM), which highlights the partial similarities and differences between LGG and GBM.
- The ncRNAs AC020907.1, Y_RNA, TMEM72‐AS1, KRT16P2, DLX6‐AS1 and AP002414.1 would competitively interact with hsa‐miR‐424, which would bind to TBPL1 mRNA, indirectly promoting TBPL1 expression.
- TBPL1 would bind to the NPAS2 promoter to promote its expression, and NPAS2 would interact with BMAL1 to promote CRY2 and PER2 expression and suppress C‐MYC expression. CRY2, which complexes with PER2 would bind to the promoters of C9ORF40, ETNK1, and VPS33B to promote their expression. Low ETNK1 expression would directly promote malignant glioma proliferation.
- VPS33B would promote the organization and release of exosomes that deliver CT62, DPY19L2P1, and KCNH1‐IT1 to immune cells, suppressing the differentiation of M1 macrophages into M2 macrophages.
- C9ORF40 would promote cellular senescence and thus inhibit tumorigenesis through p21/PCNA, p21/CDK, and p21/caspase‐3.
- The ncRNAs AC008738.2, AC102941.1, and AL136115.1 would bind to PCNA and thus inhibit cell proliferation and promote cellular senescence.

## CONFLICT OF INTEREST

The authors declare no conflicts of interest.

## AUTHOR CONTRIBUTIONS

**Yutao Huang:** Conceptualization (lead); Data curation (lead); Formal analysis (lead); Investigation (lead); Methodology (lead); Resources (lead); Software (lead); Validation (lead); Visualization (lead); Writing – original draft (lead); Writing – review & editing (lead). **Xiangyu Gao:** Investigation (equal); Methodology (equal); Resources (equal); Validation (equal). **Erwan Yang:** Investigation (equal); Methodology (equal); Resources (equal); Validation (equal). **Kangyi Yue:** Investigation (equal); Methodology (equal); Resources (equal); Validation (equal). **Yuan Cao:** Investigation (equal); Resources (equal). **Boyan Zhao:** Investigation (equal); Resources (equal). **Haofuzi Zhang:** Investigation (equal); Resources (equal). **Shuhui Dai:** Investigation (equal); Resources (equal). **Lei Zhang:** Investigation (equal); Resources (equal). **Peng Luo:** Conceptualization (equal); Formal analysis (equal); Funding acquisition (equal); Investigation (equal); Methodology (equal); Project administration (equal); Resources (equal); Software (equal); Supervision (equal); Validation (equal); Visualization (equal); Writing – review & editing (equal). **Xiaofan Jiang:** Funding acquisition (equal); Project administration (equal); Supervision (equal); Writing – review & editing (equal).

## Supporting information

Supplementary Material
