# Supplementary material for: Top‐down stepwise refinement identifies coding and noncoding RNA‐associated epigenetic regulatory maps in malignant glioma
Source: J Cell Mol Med. 2022 Feb 22;26(8):2230–50. doi: 10.1111/jcmm.17244 (PMC8995455; doi:10.1111/jcmm.17244)
Supplement: Supplementary file 1 — Supplementary Material [file JCMM-26-2230-s001.docx]

Supplementary materials for

Top-down Stepwise Refinement Identifies Coding and Noncoding RNA-Associated Epigenetic Regulatory Mappings in Malignant Glioma

Yutao Huang^1,#^, Xiangyu Gao^1,2,#^, Erwan Yang^1,#^, Kangyi Yue^1,2,#^, Yuan Cao^1^, Boyan Zhao^1^, Haofuzi Zhang^1^, Shuhui Dai^1^, Lei Zhang^1^, Peng Luo^1,^*, Xiaofan Jiang^1,^*

^1^ Department of Neurosurgery, Xijing Hospital, Fourth Military Medical University, Xi’an 710032, PR China.

^2^ State Key Laboratory of Cancer Biology, Fourth Military Medical University, Xi’an 710032, PR China.

***** Correspondence: **Peng Luo**, Department of Neurosurgery and Institue of Neurosurgery of PLA, PLA’s Key Laboratory of critical care medicine, Xijing Hospital, Fourth Military Medical University, 169 Changle Xi Road, Xi'an 710032, China. E-mail: pengluo@fmmu.edu.cn. Phone: +86-2984-775-5330, Fax: +86-2984775567. **Xiaofan Jiang**, Department of Neurosurgery and Institue of Neurosurgery of PLA, PLA’s Key Laboratory of critical care medicine, Xijing Hospital, Fourth Military Medical University, 169 Changle Xi Road, Xi'an 710032, China. E-mail: jiangxf@fmmu.edu.cn. Phone: +86-2984-775-5323, Fax: +86-2984775567.

**^#^** The authors contributed equally to this work.

**This file includes:**

Section Ⅰ: Keywords used for recognized and extracted ncRNAs.

Section Ⅱ: Supplementary Figures (S1, S2, S3) and corresponding legends.

Section Ⅲ: Supplementary Table 1, Supplementary Table 2, and corresponding legends.

SECTION Ⅰ: Keywords Used for Recognized and Extracted ncRNAs

sense_overlapping|processed_pseudogene|unprocessed_pseudogene|misc_RNA|snRNA|rRNA_pseudogene|transcribed_unprocessed_pseudogene|IG_V_gene|transcribed_processed_pseudogene|TR_V_gene|snoRNA|IG_V_pseudogene|scaRNA|TR_V_pseudogene|rRNA|pseudogene|IG_J_gene|unitary_pseudogene|transcribed_unitary_pseudogene|TR_J_gene|IG_J_pseudogene|polymorphic_pseudogene|IG_D_gene|Mt_tRNA|ribozyme|IG_C_gene|TR_J_pseudogene|TR_C_gene|IG_C_pseudogene|scRNA|Mt_rRNA|translated_processed_pseudogene|TR_D_gene|translated_unprocessed_pseudogene|sRNA|vault_RNA|lincRNA|3prime_overlapping_ncrna|processed_transcript|antisense|sense_intronic|bidirectional_promoter_lncRNA|lncRNA|TEC|macro_lncRNA|non_coding

SECTION Ⅱ: Supplementary Figures


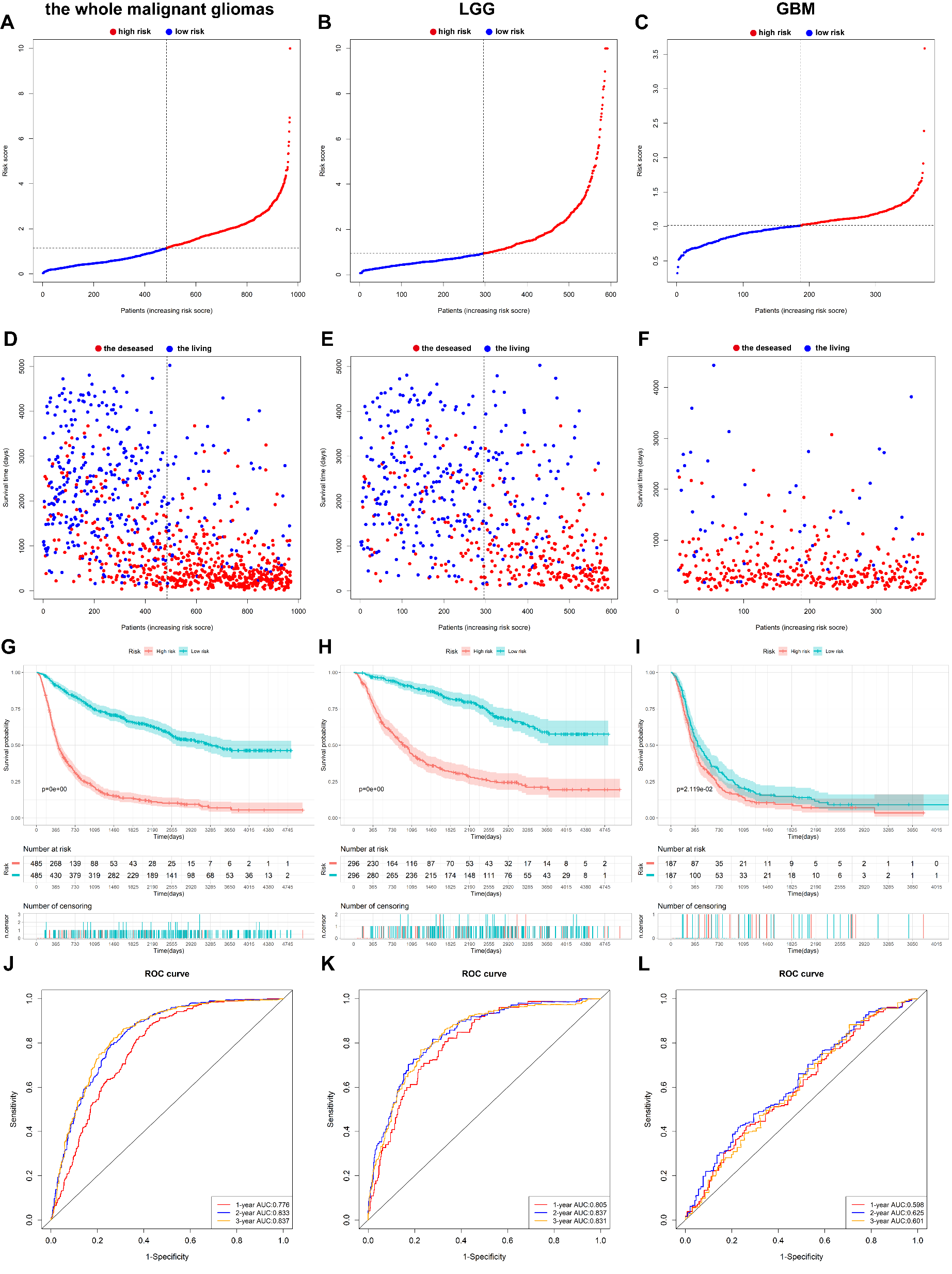


**Supplementary Figure S1. External validation of the model.** The group of **A)** the whole malignant glioma patients, **B)** the LGG patients, and C**)** the GBM patients. Each dot represents a patient; red dot, high-risk group patients; blue, low-risk group patients. Survival state of **D)** the whole glioma patients, **E)** the LGG patients, and **F)** the GBM patients; Each dot represents a patient; The dots on the left of the dashed line, low-risk group patients; the dots on the right of the dashed line, high-risk group patients; Red dots, deceased patients; blue dots, living patients. Kaplan-Meier survival analysis of **G)** the whole glioma patients, **H)** the LGG patients, and **I)** the GBM patients; Red, high-risk group patients; blue, low-risk group patients; P<10^-20^ is shown as 0. TDROC curve of **J)** the whole glioma patients, **K)** the LGG patients, and **L)** the GBM patients; Red, 1-year curve; blue, 2-year curve; orange, 3-year curve.


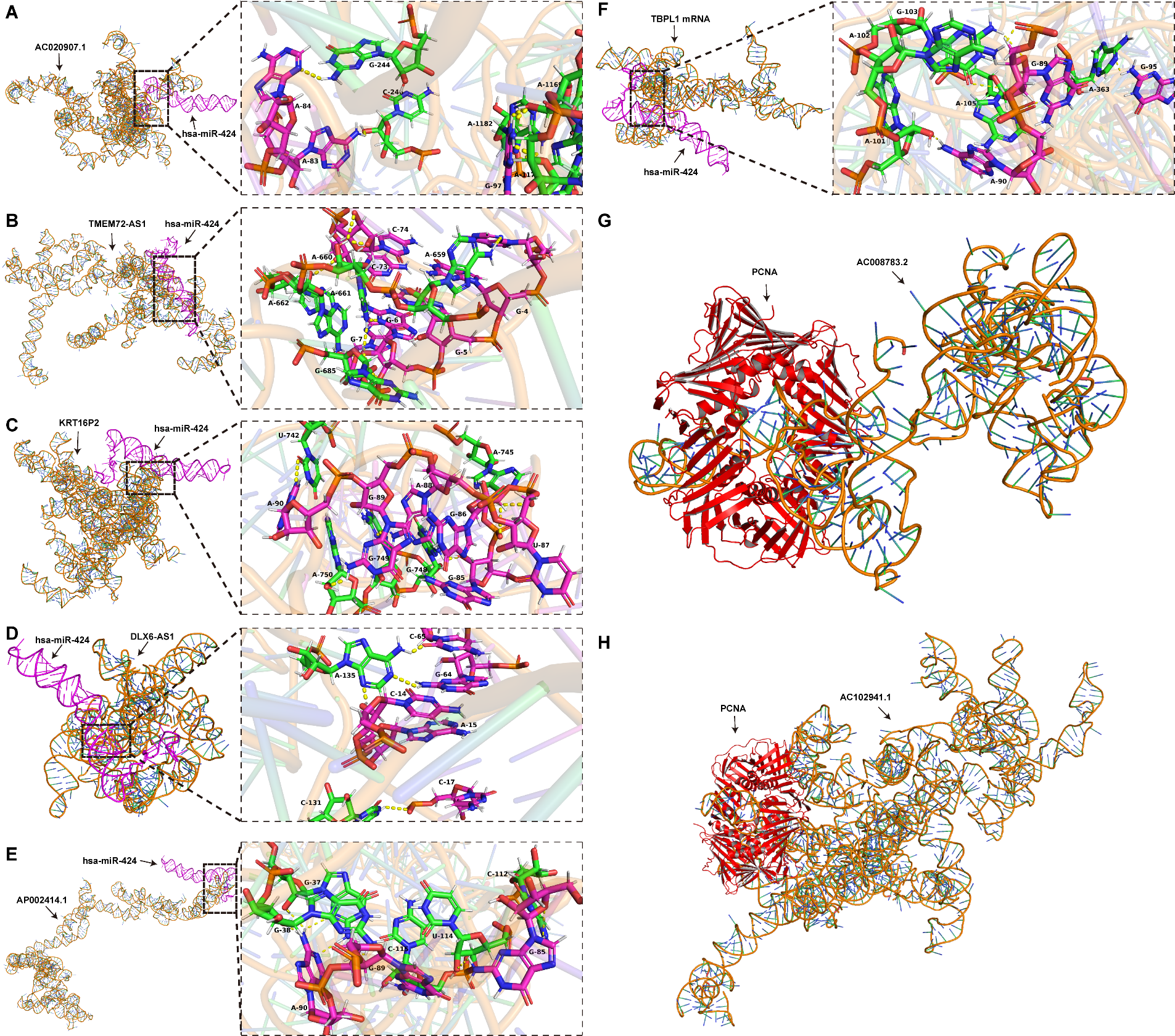


**Supplementary Figure S2. Molecular docking. A)** The interaction between AC020907.1 and hsa-miR-424. **B)** The interaction between TMEM72-AS1 and hsa-miR-424. **C)** The interaction between KRT16P2 and hsa-miR-424. **D)** The interaction between DLXS-AS1 and hsa-miR-424. **E)** The interaction between AP002414.1 and hsa-miR-424. **F)** The interaction between TBPL1 mRNA and hsa-miR-424. **G)** The interaction between the PCNA trimer and AC008783.2. AC008783.2 competitively goes through the cavity of PCNA, which hinders the DNA from going through the same site. **H)** The interaction between the PCNA trimer and AC102941.1. AC102941.1 competitively goes through the cavity of PCNA, which hinders the DNA from going through the same site.


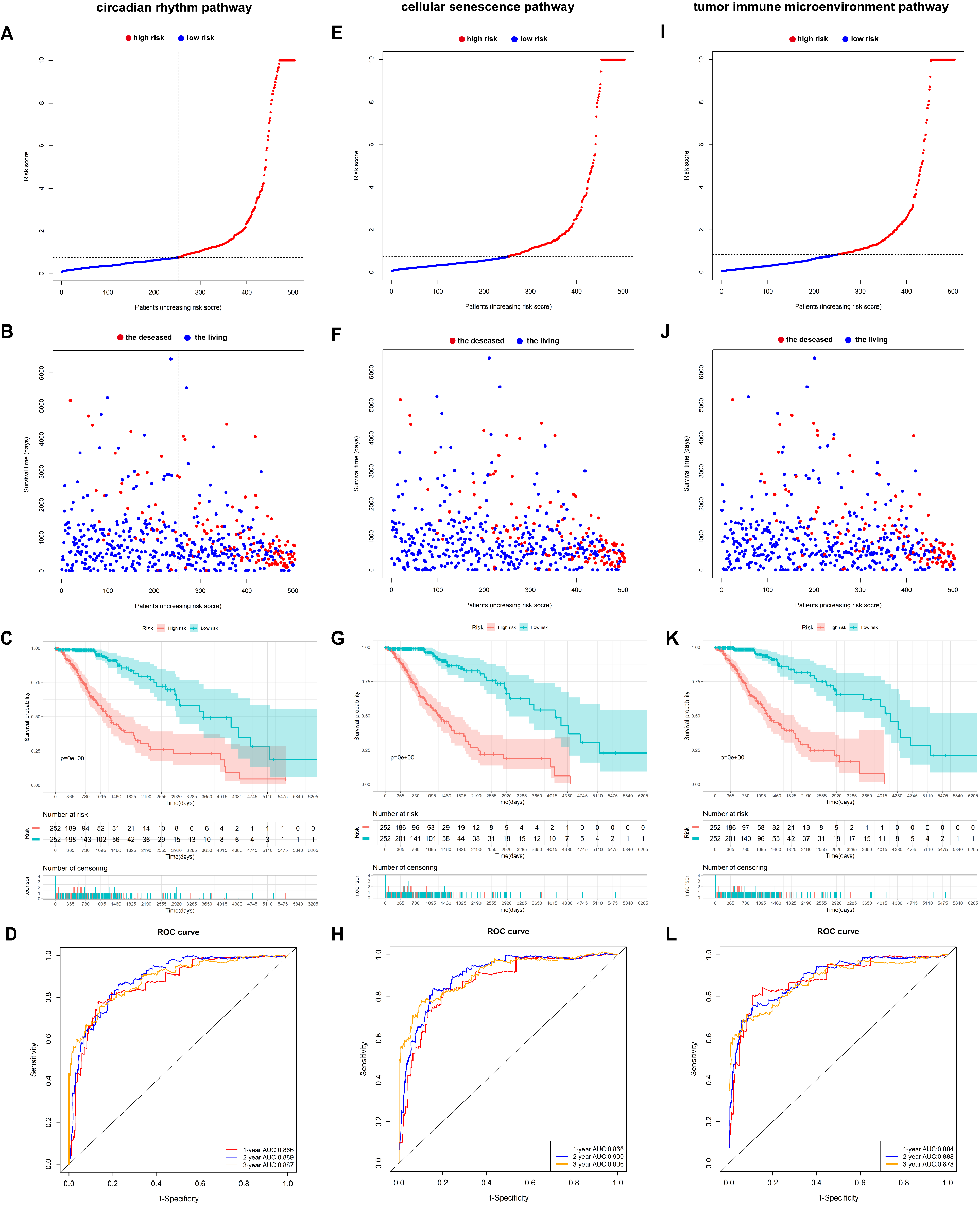


**Supplementary Figure S3. Clinical significance of the model in malignant glioma.** Patients were grouped into two groups, a high-risk group and a low-risk group, based on the expression of **A)** circadian rhythm-related genes, **E)** cellular senescence-related genes, and **I)** tumor immune microenvironment-related genes. Each dot represents a patient; red dot, patients in the high-risk group; blue, patients in the low-risk group. Survival state of patients based on the **B)** circadian rhythm pathway model, **F)** cellular senescence pathway model, and **J)** tumor immune microenvironment pathway model. Each dot represents a patient. Dots on the left of the dashed line represent patients in the low-risk group; dots on the right of the dashed line represent patients in the high-risk group; red dots, deceased patients; blue dots, living patients. Kaplan-Meier survival analysis based on the **C)** circadian rhythm pathway model, **G)** cellular senescence pathway model, and **K)** tumor immune microenvironment pathway model; red, patients in the high-risk group; blue, patients in the low-risk groups; P<10^-20^ is shown as 0. TDROC curve based on the **D)** circadian rhythm pathway model, **H)** cellular senescence pathway model, and **L)** tumor immune microenvironment pathway model; red, 1-year curve; blue, 2-year curve; orange, 3-year curve.

SECTION Ⅲ: Supplementary Tables

**Supplementary Table 1. primer sequences**

| **Name** | **Forward (5'->3')** | **Reverse (5'->3')** |
| --- | --- | --- |
| DLX6-AS1 | CACACCTGGATGTGCTCACT | CCGCAATGGCCAAAAACAGA |
| KRT16P2 | CCCTCAACAAGCCGCAATTT | CTGTTCTCCAGGGACGCTTT |
| TMEM72-AS1 | ATGTGAATGGGTGGGCAGTT | GATGCAGAGGGACGCTAACA |
| AC020907.1 | GTCCTAGCTCTTGTGCCGAG | GACGTGTGAGTGCATGGTTG |
| Y_RNA | ACCGGACTACTACCACCCAA | TCTGTCAGTTTAAATCGTCACCTCT |
| AP002414.1 | TCAACCGGGACCGAATGAAG | CGACATCGACGAGGACGAAT |
| AL136115.1 | TGGCGTAAGAAAGGAATGTGG | GGGTGATGGTAGAGATCTTGGG |
| CT62 | GAGGTCAACAACGGGATGGT | GCCTGAAGACTCCCTGACAC |
| DPY19L2P1 | TTAAAGACCCTCGTGTTGCC | TCACTCAGGCGAATGTGGTC |
| KCNH1-IT1 | TGAGGGGCAGATGGTTTTCC | CTCGCCTCTCAAGGCTTCAT |
| AC008738.2 | TATGATGGCAGCCTGCTTGGA | CAGCTCAAAGGTCTGCTCCTG |
| AC102941.1 | TCAGCTTGGATGCCTTCACC | CGGTGTGCATTGCTTCAGTT |
| hsa-miR-424 | AGGGGATACAGCAGCAATTCA | ACCTTCTACCTTCCCCACGA |
| TBPL1 | GACGCTTAGCCCGTAGTCTG | CCGATAGCACACAGCAGGAT |
| C9ORF40 | AAAATCGACGCAGGGACCAT | CACTGCTTCGGCTCCTTACA |
| NPAS2 | CACAGGAGATGGGGAGAGGA | GCTTCTCTGATTGGTGGGCT |
| VPS33B | CAGAGTGGATCAGGGAGGGA | TCCCTCTTCCTCAAGCACCA |
| ETNK1 | ACGTCACCGTTCAGGATCAG | CAAAGCCCACAATCCCCAAAA |
| CRY2 | GCTGCGACTCCACGACAAC | AAACAGGCGGGAGTTCAGTT |
| GAPDH | CAATGACCCCTTCATTGACC | GACAAGCTTCCCGTTCTCAG |
| U6 | CTCGCTTCGGCAGCACA | AACGCTTCACGAATTTGCGT |

**Supplementary Table 2. Transcription factor-binding sites.**

| **Transcription factor** | **Gene** | **Start** | **Stop** | **Strand** | **Binding site sequence** | **Relative score** |
| --- | --- | --- | --- | --- | --- | --- |
| TBPL1 | NPAS2 | -1304 | -1297 | + | TGACAGTT | 0.96 |
| TBPL1 | NPAS2 | -751 | -744 | + | TTATGGTT | 0.92 |
| TBPL1 | NPAS2 | -544 | -537 | - | AAACAGTT | 0.92 |
| TBPL1 | NPAS2 | -1539 | -1532 | + | TGACTGTT | 0.91 |
| NR4A2 | C9ORF40 | -1389 | -1382 | - | GAGGTCAG | 0.90 |
| NR4A2 | ETNK1 | -366 | -359 | + | AAGATCAA | 0.90 |
| NR4A2 | ETNK1 | -1300 | -1293 | + | GAGGTCAG | 0.90 |
| NR4A2 | VPS33B | -432 | -425 | + | AAGGTCAC | 1.00 |
| NR4A2 | VPS33B | -1773 | -1766 | + | GAGGTCAA | 0.92 |
| NR4A2 | VPS33B | -873 | -866 | + | GAGGTCAA | 0.92 |
| NR4A2 | VPS33B | -1225 | -1219 | - | GAGGTCAG | 0.90 |

Negative numbers represent the number of bases upstream of the transcription start site where the binding site is located.
